# Factors associated with the outcomes of a novel virtual reality therapy for military veterans with PTSD: Theory development using a mixed methods analysis

**DOI:** 10.1371/journal.pone.0285763

**Published:** 2023-05-25

**Authors:** Ben Hannigan, Robert van Deursen, Kali Barawi, Neil Kitchiner, Jonathan I. Bisson

**Affiliations:** 1 School of Healthcare Sciences, Cardiff University, Cardiff, United Kingdom; 2 School of Medicine, Cardiff University, Cardiff, United Kingdom; 3 Veterans’ NHS Wales, Cardiff and Vale University Health Board, Cardiff, United Kingdom; Mokpo National University, REPUBLIC OF KOREA

## Abstract

**Background:**

Multi-modular motion-assisted memory desensitization and reconsolidation therapy (3MDR) is a new psychological intervention for people with post-traumatic stress disorder (PTSD). 3MDR is immersive, delivered in a virtual reality environment, and emphasises engagement, recollection and reprocessing.

**Objective:**

Through a theory-driven examination of data relating to 10 out of 42 UK military veterans taking part in a trial of 3MDR, the principal objective was to explore the complex interrelationships between people, interventions and context and to investigate how factors within these domains interacted in specific outcome typologies.

**Method:**

Quantitative and qualitative data relating to 10 trial participants were derived from: researcher-assessed and self-report clinical measures; interviews; physiological recordings; words describing thoughts and feelings during therapy; and subjective unit of distress scores. Using a convergent mixed methods approach, data were tabulated using a person, intervention and context model. Participant summaries were grouped into outcome typologies, followed by an analysis of data convergence and divergence within each and an interpretation of identified patterns.

**Results:**

Three outcome response typologies were identified: dramatic improvement, moderate improvement and minimal improvement. Within the person domain, factors associated with outcomes included walking capacity, commitment and ability to complete therapy, and levels of subjective distress. Within the intervention domain, factors associated with outcomes related to image selection and use, therapeutic alliance and orientations towards the tailoring of sessions. Within the context domain, factors associated with outcomes included reactions to the therapy environment. The patterning of secondary outcomes broadly corresponded with primary outcomes within each typology. Alongside patterned data differentiating aspects of the person, intervention and context domains, within the three response typologies data also existed where no obvious patterning was detected.

**Conclusions:**

The model developed here may have novel value in evaluating a range of personalised interventions, but further work is needed before confident assertions can be made of who is likely to benefit from 3MDR specifically.

## Introduction

People with post-traumatic stress disorder (PTSD) often live with a range of distressing symptoms and related functional impairments, which can include re-experiencing, hyperarousal, dissociation, changes in mood and cognition, and more [1]. Lifetime prevalence of PTSD has been estimated at 6.8% [2], with a recent systematic review of the evidence supporting the use of trauma-focused cognitive behavioural therapies along with eye movement desensitization and reprocessing (EMDR) [3]. In the UK, specific interventions recommended by the National Institute for Health and Care Excellence (NICE) include cognitive processing therapy, cognitive behavioural therapy with a trauma focus and narrative exposure therapy, with EMDR indicated in some circumstances but not for adults with combat-related trauma due to the lack of sufficient evidence [1]. It is known, however, that not all people living with PTSD and in receipt of evidence-based therapy obtain relief from their distressing symptoms, and that significant numbers of military veterans with PTSD either do not engage with or drop out of therapy [4]. A further recent systematic review has concluded that relatively little is known about what helps and hinders progress with treatment [5].

Uncertainty over the factors associated with treatment progress is not limited to the case of PTSD, with the more general observation being made that it is often not obvious why any psychological therapy succeeds or fails [6]. This is important, as without this understanding it is difficult to know what individualised modifications are needed in order to optimise effectiveness [7, 8]. Interest is therefore growing in moving beyond mechanistic approaches to the delivery and evaluation of mental health interventions, including in ways which reflect a wider health and social scientific concern with the principles of complexity, variation and interconnectedness [9]. In this context, two pressing research challenges arise. First, is the development and evaluation of novel, tailored, interventions for people not benefiting from current best-evidence therapies. Second, is the granular examination of factors associated with both favourable and less-favourable therapy outcomes. This paper makes a distinct contribution in both these areas. It is underpinned by an awareness of psychological therapies as always being complex interventions, in which complexity arises because of the properties of (and the interplay between) the therapy itself and the wider environment within which this is delivered [10, 11]. Through the specific case of a novel therapy for people with PTSD our aim in this paper is to advance understanding of the interrelationships between people, interventions and context and to explore how factors within these domains interact, including in specific typologies of outcome trajectory.

## Building a theoretical model: 3MDR as a complex intervention for people with complex difficulties

**Fig 1:**
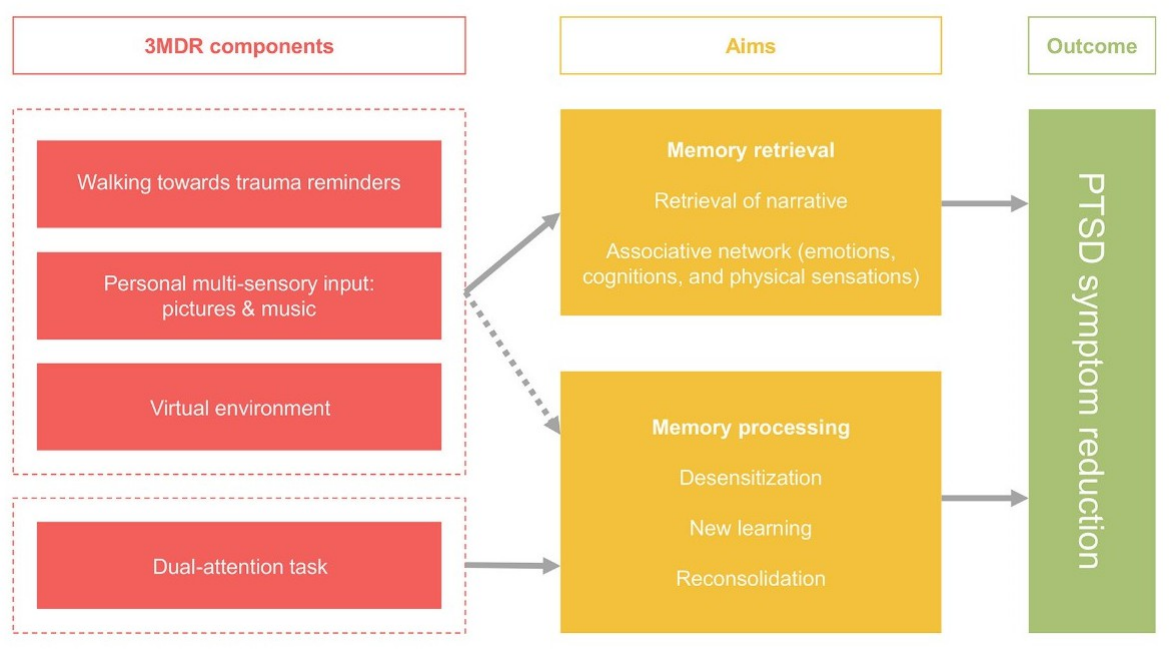
Components, aims and outcomes in 3MDR [reproduced using a Creative Commons CC-BY licence from van Gelderen et al. 2018, 17].

Multi-modular motion-assisted memory desensitization and reconsolidation therapy (3MDR) is an immersive psychological intervention first developed for military veterans with combat-related PTSD [12]. It is one of a number of immersive therapies which have recently appeared within the field of health care, with other examples including virtual reality interventions to enhance treatments for psychosis [13], improve neurocognitive functioning following brain injury [14], aid rehabilitation after stroke [15], and reduce stress in people with multiple sclerosis [16]. In 3MDR specifically, the person with PTSD walks on a treadmill towards a series of immersive, personally selected, images displayed on a wide screen and interacts with these with support from a therapist. The therapy is informed by theory, which emphasises: the particular value of a virtual reality environment in maximising engagement and presence; the importance of walking towards symbolic representations of previous trauma, rather than avoiding past, painful, memories; the use of multi-sensory stimuli (images and music) to directly connect the person to their trauma and to promote recollection and narration; and the application of dual-attention tasks to tax working memory, leading to the processing and reconsolidation of newly adjusted recollections [17]. Fig 1 below, reproduced directly from van Gelderen et al.’s [17] overview of theory and research underpinning 3MDR, summarises the relationships between this new therapy’s constituent components, its aims and intended outcomes:

3MDR therefore comprises multiple moving parts (literally, given the use of a treadmill on which the person with trauma walks) which, delivered by therapists and received by patients interacting together in particular contexts, are thought to trigger cognitive and emotional change. However, whilst it benefits from a theoretical base and emerging empirical evidence of its effectiveness [18–20], the combination and sequencing of critical ingredients that result in 3MDR being (or not being) a beneficial therapy are not self-evident. In this context, in their systematic review of factors associated with outcomes of psychological therapies for people with PTSD, Barawi et al. [5] make a case for greater research attention to be paid to the full range of personal, clinical, social, economic and contextual factors with a possible bearing on patient response. As a response to this call, further disaggregation of 3MDR beyond the elements summarised in Fig 1 above reveals a number of other components which may also contribute to its effect. These include: the personal characteristics and background of the individual receiving 3MDR; the nature of their trauma and their response to it; the extent to which the person living with trauma is socially supported, and is encouraged to participate in therapy; their expectations and motivations; the practicable ability of the person to make repeated journeys to a technically specified and suitably equipped 3MDR clinic; the processes through which multi-sensory stimuli are selected, and the manner in which trauma-associated images are sequenced both within and between therapy sessions; individual therapist interactional style and therapeutic alliance, which even in a standardised intervention tested in controlled conditions will vary; the physical, as well as the cognitive and emotional, response of the person during therapy sessions; and participation in a novel therapy where the most helpful and acceptable number of weekly sessions is not known in advance. 3MDR is also manifestly not a passive intervention acting independently of the person receiving it; rather, he or she has agency, engaging in therapy co-creation and in the co-production of outcomes.

**Fig 2:**
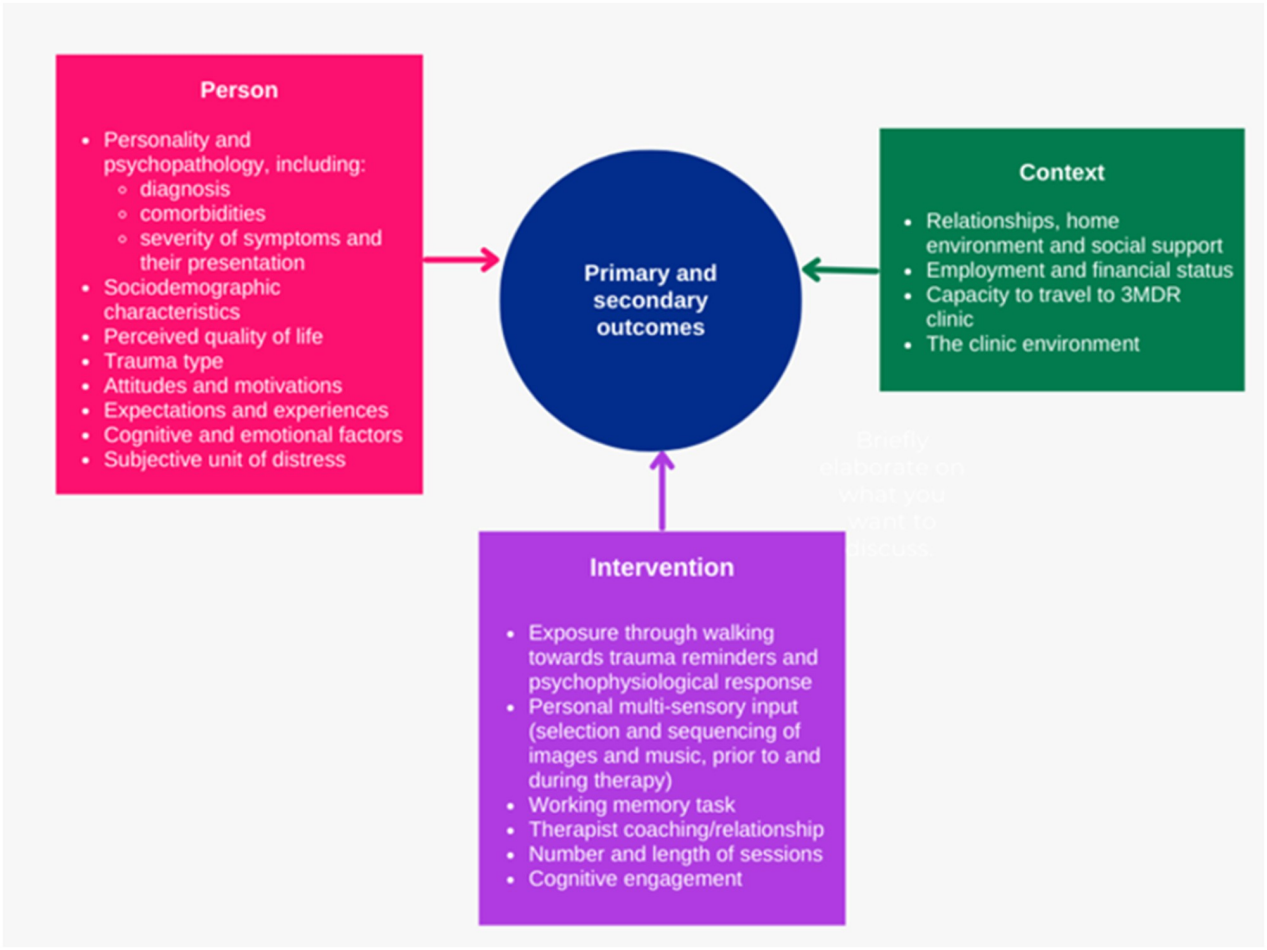
Factors associated with the outcomes of 3MDR therapy.

In Fig 2 below, we propose a three-domain model which brings these elements together and offers a heuristic frame through which therapy outcomes might be explored and better understood. The components in the ‘Intervention’ domain include, and expand on, the components identified in the model proposed by van Gelderen et al. [17] above and also reflect the broader ‘aspects of treatment’ dimension identified in Barawi et al.’s systematic review of factors associated with outcomes [5].

## Material and methods

**Fig 3:**
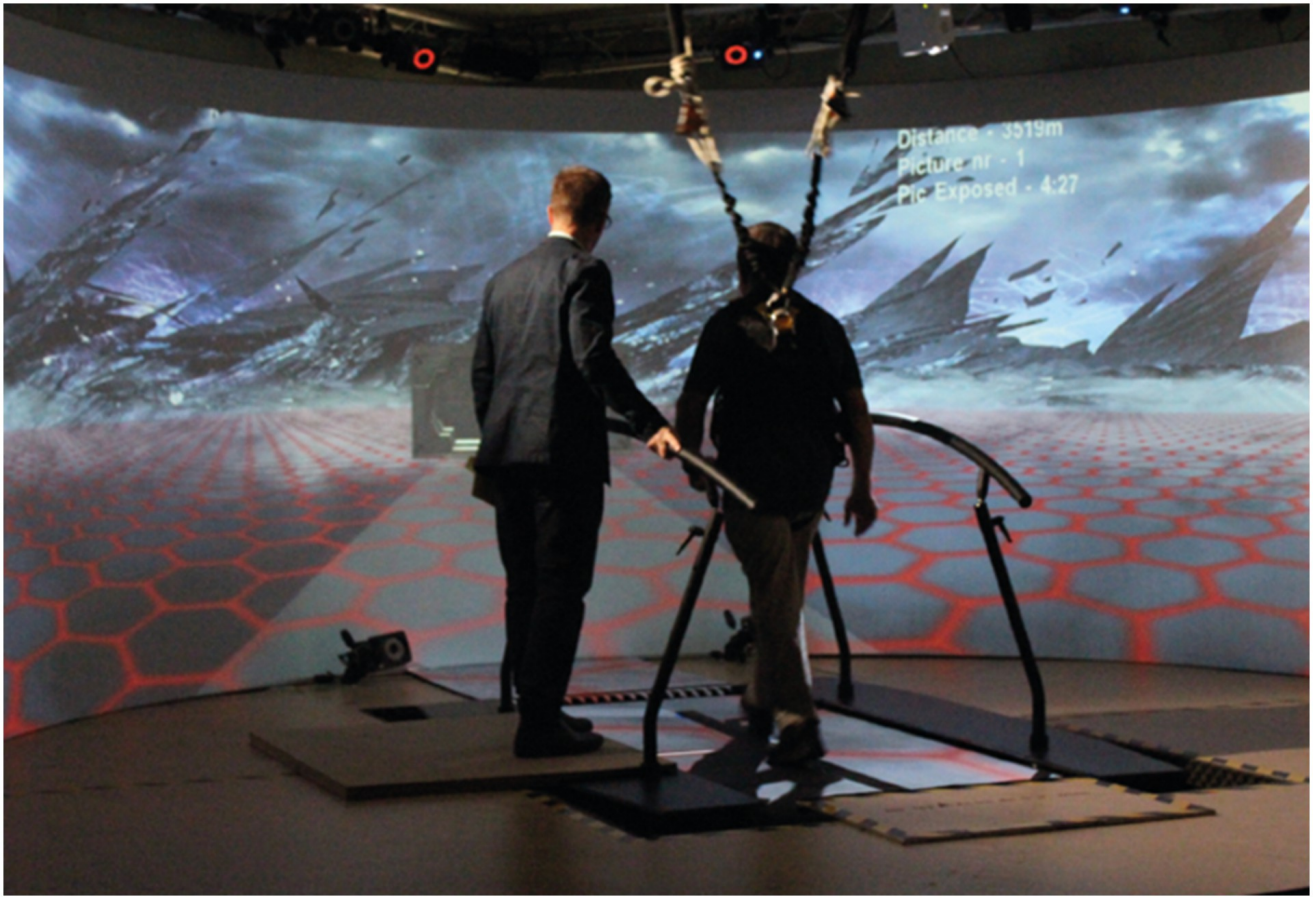
3MDR session [image reproduced from Bisson et al. 2020, 21].

The data used in this paper are drawn from a recently completed phase II randomised controlled trial of 3MDR for UK military veterans with treatment-resistant PTSD [Trial Registration Number: ISRCTN80028105] [19]. Ethical approval for the study was granted by the South East Wales Research Ethics Committee (reference 17/WA/0005), and all participants gave written informed consent. The 42 male participants were randomised to immediate, or 12 week delayed, treatment arms. During active treatment (following an initial period of preparation) participants were offered six weekly sessions of 3MDR and then a single concluding session. 3MDR therapy sessions were provided in a Motek Gait Real-time Analysis Interactive Lab (GRAIL™), complete with treadmill and motion-capture, virtual reality, system (see Fig 3 below). During therapy, participants were attached to a Zephyr™ BioHarness™ which both ensured safety and supported real-time recordings of breathing rate (BRT) and heart rate (HRT). Reflecting 3MDR theory [17], each therapy session opened and closed with the playing of participant-selected music, the first a piece associated with the period of trauma ahead of the person with PTSD walking, on the treadmill and in the company of a skilled psychological trauma therapist specifically trained in 3MDR, towards a sequence of seven participant-selected images displayed on a large virtual reality projector screen. Each image was associated with the participant’s past trauma, and for each image in turn participants were asked to describe thoughts, feelings and physical sensations. The words used were displayed on the projector screen, and recorded to become part of the data corpus, ahead of the dual task exercise designed to tax working memory. This was triggered by a red ball moving across the screen, with participants asked to continue focusing on their displayed words and phrases whilst tracking the ball’s motion and naming the random numbers displayed on it until both the ball and the phrases disappeared after approximately 30 seconds. A final part of each image cycle was the voicing and recording of a score (using a 0–10 scale) to represent a subjective unit of distress (SUD). Each session concluded (ahead of a therapist debrief) with the participant walking to the sound of their second piece of music associated with the here-and-now.

Standardised clinical, psychological and social assessments were made using validated measures, and were completed at three key points (baseline, and at weeks 12 and 26) by members of the project team blinded to treatment condition. The measures used within the main trial yielding data drawn on in this paper are summarised below, with more detail to be found in Bisson et al. [19]:

1. The Clinician Administered Post Traumatic Stress Scale (CAPS-5) [22]. This is considered the gold standard measure for assessing PTSD and was used as the trial’s primary outcome measure. It comprises 30 semi-structured interview questions, which aim to measure PTSD symptoms across a lifetime, one month or one week and provides PTSD severity and frequency of symptoms.
2. The Life Events Checklist for DSM-5 (LEC-5) is a self-report measuring which is designed to be used before administering the CAPS-5 assessment [23]. The LEC-5 explores 16 traumatic events and includes one additional item assessing any other distressing event not captured in the 16 items. There is no formal scoring for the LEC-5 as it is intended to gather information on the individual past life experiences. Individuals indicate varying levels of exposure to each potentially traumatic event on a six-point scale. The individual can list multiple levels of exposure to the same trauma.
3. The Patient Health Questionnaire (PHQ-9) is a well-validated and reliable brief self-report measure of depression [24]. Scores represent: 0–5 = mild; 6–10 = moderate; 11–15 = moderately severe; 16–20 = severe depression.
4. The Generalised Anxiety Disorder (GAD-7) is a widely used, reliable, and a well-validated brief self-report measure of anxiety [25]. Scoring can be calculated by assigning 0,1,2 and 3 to the categories “not at all,” “several days,” “more than half the days,” and “nearly every day,” respectively. Total scores range from 0–21 and represent: 0–5 mild; 6–10 moderate; 11–15 moderately severe anxiety; 16–21 severe anxiety.
5. The Multidimensional Scale for Perceived Social Support (MSPSS) comprises 12 questions that measure family, friends and partner support [26]. The reliability and validity of the MSPSS have been shown with several populations. One of the methods to categorise the scoring divides responses into three equal groups based on their scores and allocating the lowest group as ‘low perceived support’, the middle group as ‘middle perceived support’ and the highest group as ‘high support’. The calculation for this group is as follows; 1–24 (lowest perceived support); 25–48 (medium); 49–72 (high).
6. The Work and Social Adjustment Scale (WSAS) is a self-report measure that assesses the individual’s mental health difficulties with work, social activities, and personal or family relationships [27]. The maximum score of the WSAS is 40, where lower scores indicate better functioning. Scores below 10 appear to be associated with subclinical populations. A score between 10 and 20 is associated with significant functional impairment but less severe clinical symptomatology. A score of 21 or more suggest severe or worse psychopathology.
7. The EuroQol five-dimensional descriptive system (EQ-5D-5L) is an instrument in health economic analysis that measures health-related quality of life [28]. The first part of the questionnaires identified problems five areas of physical health; mobility, self-care; usual activities; pain and discomfort; and anxiety and depression. The response to each questionnaire is rated on a 1–5 scale, which represents: 1 = no problems; 2 = slight problems; 3 = moderate problems; 4 = severe problems; and 5 = extreme problems. The second part is a visual scale, which measures the current health status on a 0–100 scale.

Principal outcomes from the trial described above been reported by Bisson et al. [19], and these reveal 3MDR to be a promising intervention. As part of the trial a nested process evaluation was also conducted, through which (following the completion of their therapy) 11 purposively sampled veterans were interviewed using a semi-structured schedule. Participant selection for post-intervention interviews was designed to ensure maximal variation, with the target number of around 10 interviewees reflecting the need for a sufficiently heterogeneous sample with whom detailed, in-depth, data could be generated [29]. Within the final group of 11 taking part in post-therapy interviewees were: at least one participant treated by each of the six therapists; participants who, to their therapists, appeared to have benefited from therapy; and participants who appeared not to have benefited or had dropped out. Audio-recorded interviews covered participants’ motivations and expectations at the start of therapy, their experiences of 3MDR, and their reflections on its effect. Of the 11 interviewees, one had dropped out before a first therapy session had taken place, meaning that no intervention-domain data could be collected. As the analysis developed in this paper necessarily draws on data of this type, having had no therapy sessions involving exposure to traumatic images this person’s data are entirely omitted from this paper. Their data are, however, included in the main findings paper previously published from the larger trial [19]. Information on the characteristics of each of the 10 veterans whose data used in this paper are given in Table 1 below.

**Table 1:** Sample summary.

Participant ID	Age on recruitment to trial	Arm of study	Therapist ID	Reason for sampling	Length of interview (minutes)
P1	66	Delayed treatment	Therapist 1	First veteran into trial, traumas dating from the 1970s	63
P2	38	Immediate treatment	Therapist 2	First veteran to have 3MDR, traumas from the 2000s	33
P4	49	Immediate treatment	Therapist 3	Trauma not service-related	25
P8	58	Delayed treatment	Therapist 4	Multiple traumas, developmental and learning problems	94
P9	42	Immediate treatment	Therapist 5	Reported major benefits before end of scheduled 3MDR sessions, and early discontinuation	35
P10	45	Delayed treatment	Therapist 4	Strong emotional responses to therapy	47
P12	55	Delayed treatment	Therapist 6	Reported unanticipated physical health improvements	31
P18	53	Delayed treatment	Therapist 4	Co-existing physical health problems and disabilities, financial issues	53
P25	55	Immediate treatment	Therapist 4	Discontinued before final treatment session, as could not find photos evoking strong-enough emotions	48
P26	56	Immediate treatment	Therapist 1	Dropped out early	43

Taken together the dataset for these 10 participants is unusually rich, comprising: standardised researcher-assessed and self-report clinical measures; findings from post-therapy semi-structured interviews focusing on views and experiences; within-session, real-time, physiological recordings (HRT, BRT and walking pace); within-session words and phrases describing thoughts and feelings at the end of each image cycle; and SUD scores. To develop the data analysis and synthesis presented here, a four-step convergent mixed methods framework was used [29]. This approach is characterised by different but complementary types of data being pooled only once these have been independently generated and analysed. Step one involved the collection of the different types of data, as described above, with step two involving a series of separate analyses. Clinical outcomes were first described at each of two key assessment points for each of the 10 participants: immediately preceding commencement of 3MDR, and completion of a final therapy session. As the focus of this paper is on examining interactions between person, intervention and context during the period of active therapy, outcome scores were included only as these were recorded immediately prior to the commencement of therapy and immediately following its conclusion. This means that outcome scores were not included for participants in the immediate treatment arm of the trial beyond week 12 (when therapy was scheduled to have completed) and were not plotted at baseline for participants in the delayed treatment arm (this being 12 weeks prior to actual therapy commencing).

Where proposed in our model in Fig 2, baseline data were reported for all 10 participants where these contributed to our understanding of the ‘person’ domain. Examples are data on trauma experiences collected using the LEC-5, and data on comorbidity made possible through the gathering of baseline information on indicative depression and anxiety using the PHQ-9 and the GAD-7. Within and between-session physiological measures were summarised, describing each participant’s BRT, HRT and walking pace. Reflecting the model summarised in Fig 2, some types of data were used in multiple ways, with BRT serving as an example. This was included as one part of an assessment of participants’ ‘exposure through walking towards trauma reminders and psychophysiological response’, but also as a proxy for ‘cognitive engagement’ both within the ‘intervention’ domain. This decision reflected observations in this area arising from earlier 3MDR research [17] and evidence previously reported from the current trial [30]. The words and phrases used by each participant at the end of each image cycle were brought together in a single list, comprising 757 individual items. Each word or phrase was independently themed by three members of the project team (with disagreements resolved through discussion) into one of a number of categories, including ‘anxiety’, ‘depression’, ‘positivity’ and ‘negativity’. For each participant the proportion of words and phrases used in each category was calculated, both within and across sessions, and each category was further grouped into a larger ‘positive’ or ‘negative’ class reflecting overarching valence. All interviews were transcribed in full, and managed and analysed with the help of the qualitative data analysis software package NVivo (version 12) [31]. This analysis involved the production of within-case [32] summaries condensing the detail of each interview conducted with each of the 10 participants.

With each type of data subjected to this initial analysis, step three in our mixed methods approach [29] involved the bringing together of these analyses for the purposes of comparison and contrast. Condensed summaries of each separate analysis completed in step two for each participant were tabulated using the three domains represented in Fig 2 above (person, intervention and context). This step is summarised in Table 2 below.

**Table 2:** Domains and data.

Domain	Data
Person	Personality and psychopathology, including: diagnosis *Extracted from inclusion criteria data* comorbidities *Extracted from baseline scores on the Patient Health Questionnaire (PHQ-9) for indicative depression and from baseline scores on the Generalised Anxiety Disorder Questionnaire (GAD-7) for indicative anxiety* severity of symptoms and their presentation [CAPS at baseline] *Extracted from baseline scores on the Clinician Administered Post Traumatic Stress Scale (CAPS-5)* Sociodemographic characteristics *Extracted from demographic questionnaires* Perceived quality of life *Derived from baseline scores on the EuroQol-5 Dimension scale (EQ-5D-5L*, *but excluding scores on depression and anxiety given the availability of scores on the PHQ-9 and the GAD-7 as described above)* Trauma type *Derived from baseline scores on the Life Events Checklist Scale (LEC-5)* Attitudes and motivations *Derived from qualitative interviews* Expectations and experiences *Derived from qualitative interviews* Cognitive and emotional factors (both positive and negative: e.g., relief, anxiety, anger, guilt) *Extracted from words and phrases spoken by participants within sessions*, *and displayed on the video screen* Subjective unit of distress *Rated by participants on a scale of 0–10 at the end of each image cycle*
Intervention	Exposure through walking towards trauma reminders and psychophysiological response *Derived from recordings of breathing rate*, *heart rate and walking pace* Personal multi-sensory input (selection and sequencing of images and music, prior to and during therapy) *Derived from qualitative interviews* Working memory task *Subjective unit of distress*, *rated by participants on a scale of 0–10 at the end of each image cycle*, *used as a proxy for more direct measures of working memory task* Therapist coaching/relationship *Derived from qualitative interviews* Number and length of sessions *Derived from qualitative interviews* Cognitive engagement *Derived from recordings of breathing rate*, *as a proxy for more direct measures of cognitive engagement*
Context	Relationships, home environment and social support *Derived from baseline scores on the Multidimensional Scale for Perceived Social Support scale (MSPSS)* *Derived from qualitative interviews* Employment and financial status *Derived from baseline scores on the Work and Social Adjustment scale (WSAS)* *Derived from qualitative interviews* Capacity to travel to 3MDR clinic *Derived from qualitative interviews* The clinic environment *Derived from qualitative interviews*
Primary outcome and response typology, and secondary outcomes	Primary outcome and response typology *Derived from changes recorded over time in scores on the 3MDR trial’s primary outcome measure*: *the Clinician Administered PTSD Scale for DSM5 (CAPS-5)* Secondary outcomes *Derived from outcomes on selected secondary measures*: *depression (PHQ-9); anxiety (GAD-7); social support (MSPSS); work and social adjustment (WASA); quality of life (EQ-5D-5L)*

The fourth and final step involved an interpretative exploration of the condensed data brought together for the 10 participants. This was accomplished through an analysis of the extent to which data for each converged and diverged, followed by the advancement of plausible explanations for any patterns identified and described.

## Findings

**Fig 4:**
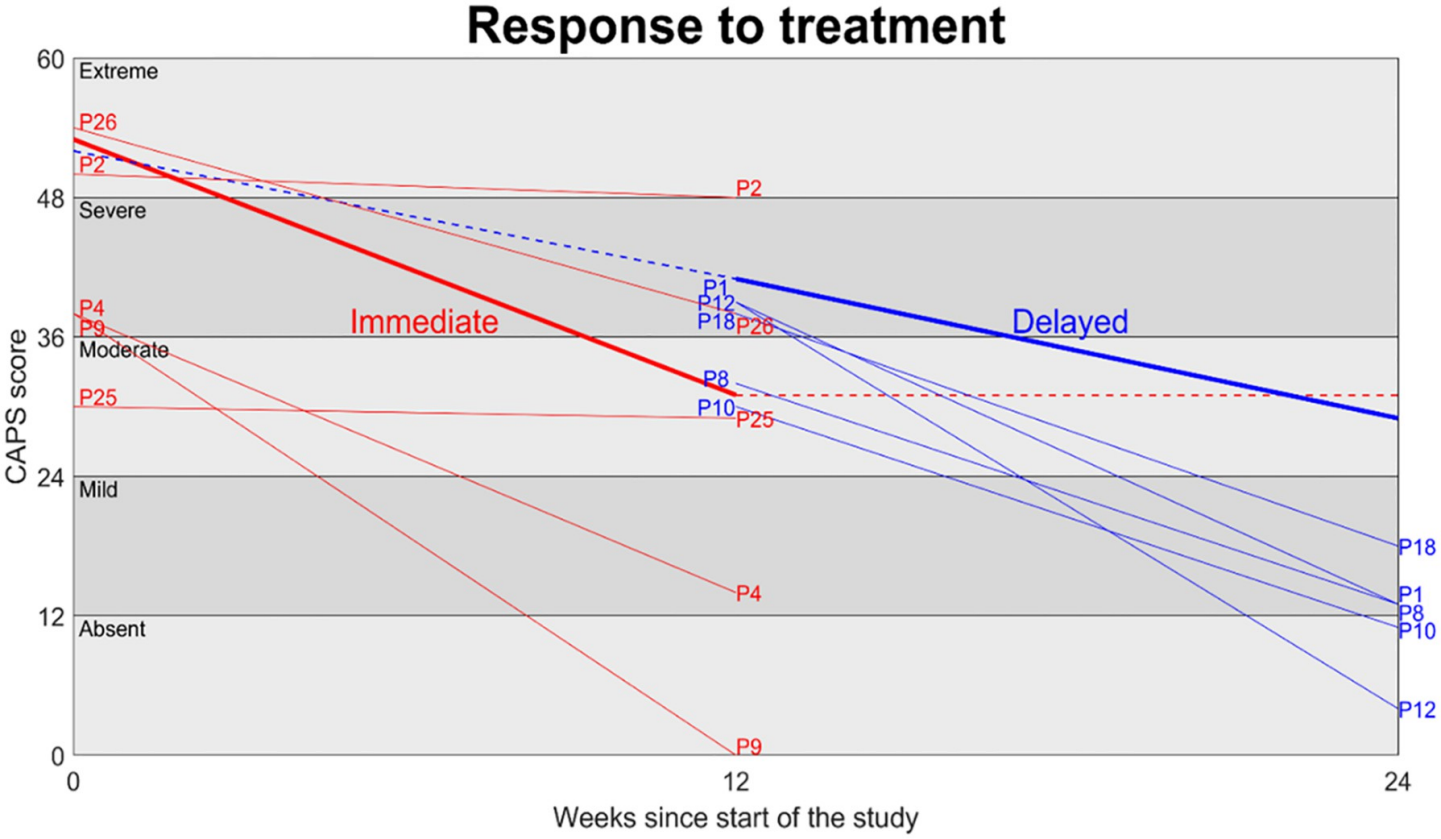
Response to treatment: CAPS-5 clinical outcomes for 10 3MDR participants for whom combinations of outcome, psychophysiological and qualitative data were gathered. Average results at the group level are shown as the thicker lines. The immediate group displayed in red received 3MDR immediately after being entered into the study. The delayed group displayed in blue acted as the control group for the first 12 weeks before receiving the intervention. The thinner lines represent the individual change in CAPS-5 score before and after the intervention. Individual participants are indicated by the labels starting with P. Individual scores are shown for the intervention periods only.

Primary outcome scores using the CAPS-5, at each of the two key assessment points for each of the 10 participants from whom combinations of outcome, psychophysiological and qualitative data were gathered, are displayed in Fig 4 below. Higher scores indicate higher levels of PTSD symptomatology.

We used these CAPS-5 primary outcome data to differentiate three distinct typologies of response to 3MDR. These typologies recognised that whilst this novel psychological therapy is promising, not all participants responded to, or benefited from, it in equal degree. Judgments on the allocation of participants to response types were made through discussion leading to consensus, drawing on the degree of change in CAPS-5 scores during periods of active therapy, and the extent to which participants moved (or did not move) between PTSD symptom severity bands as plotted on the y axis in Fig 4 and labelled from ‘extreme’ through to ‘absent’. The typologies are:

- Dramatic improvement (participants: P1, P4, P9, P12)
- Moderate improvement (participants: P8, P10, P18, P26)
- Minimal improvement (participants: P2, P25)

Two of the four participants in the dramatic improvement group moved between three PTSD symptom severity bands (from ‘severe’ to ‘absent’), and the remaining two moved between two bands and also had reductions in CAPS-5 scores of more than 20 points. The four moderate improvers moved between either one or two symptom severity bands, and had reductions in CAPS-5 scores of 20 points or less, whilst the two minimal improvers remained in the same symptom severity bands after therapy and had very small reductions in CAPS-5 scores. Scores for all participants are reported in detail in the S1 File.

Informed by the model described in Fig 2 above, and by the use of primary outcome (CAPS-5) data to distinguish different responses to therapy, the analysis completed in step 3 (involving the pooling of quantitative and qualitative data for each participant, grouped into typology) is reported in S1 File. In this we plot, in granular detail, person, intervention, context and outcomes for all 10 participants for whom complete combinations of clinical, psychophysiological and qualitative data are available. In Table 3 below, arising from step 4 in our mixed methods approach, we summarise these data within each of the three response typologies to inform the identification of typology-specific patterns.

**Table 3:** Person, intervention, context and outcomes for 10 participants grouped within three response typologies.

Response typology	Person	Intervention	Context	Secondary outcomes
Dramatic improvement P1 (delayed arm) P4 (immediate arm) P9 (immediate arm) P12 (delayed arm)	***Personality and psychopathology*** All participants living with PTSD, all had previous trauma-focused psychological treatment without loss of PTSD diagnosis. Three of the four had coexisting depression and anxiety. All four had severe PTSD symptoms. ***Sociodemographic characteristics*** Aged between 42–66. One educated to degree level or above, one with an apprenticeship and two with school-leaver qualifications. ***Perceived quality of life*** None of the participants had problems walking about, washing or dressing themselves. Two had no problems undertaking their usual activities, whilst two had moderate problems. All participants had different scores on pain/discomfort, from none to severe. One participant felt their health was optimal on the day, whilst two scored below population average and one scored substantially lower. ***Trauma type*** All four experienced severe trauma. ***Attitudes and motivations*** Participants motivated by opportunity to alleviate own symptoms, try a new therapy, and by prospect of helping others. ***Expectations and experiences*** Some apprehension and uncertainty, all four described 3MDR as highly immersive. ***Cognitive and emotional factors*** Overall, negative valence in choice of phrases was uniformly found to be very predominant in this group. Specifically, anxiety was predominant followed by anger and depression. Phrases with positive valence accounted for approximately 20% of all phrases used. ***Subjective unit of distress*** Subjective distress varied within the group. Two of the four started with extreme/severe levels of distress which dropped rapidly to absent levels at the end. One started with extreme distress which gradually dropped to severe at the end. The fourth started with mild distress, this gradually rising to moderate at the end.	***Exposure through walking towards trauma reminders and psychophysiological response*** All four walked relatively slowly, imposing relatively low physical demand. All four had at least one treatment session in which they consistently demonstrated a very rapid breathing rate. For two this applied to all their sessions. For the other two, initial sessions generated a low breathing response. Three out of the four consistently demonstrated moderately raised heart rates reflective of low physical demand. One demonstrated a minimally raised heart rate barely above the resting rate. Stronger heart rate responses were expected in this context. ***Personal multi-sensory input (selection and sequencing of images and music)*** Finding images a hard task for all four, involving time and effort, sometimes traumatic and distressing. ***Working memory task*** All participants engaged in multiple tasks designed to tax working memory, by combining walking, visual tracking of a ball, calling out random numbers and scoring their subjective unit of distress at the end of each image cycle. No specific measures of demand on working memory were available. ***Therapist coaching/relationship*** Therapy either with a familiar therapist and/or with a therapist who put at ease or was trusted. ***Number and length of sessions*** Three of the four committed to completing the course, the fourth ending early as dramatic improvement reported. One would have had additional therapy. ***Cognitive engagement*** All four had at least one treatment session in which they consistently demonstrated a very rapid breathing rate. For two this applied to all their sessions. For the other two, initial sessions generated a low breathing response. Very high breathing rates could indicate cognitive engagement. No specific measures of cognitive engagement were available.	***Relationships*, *home environment and social support*** No pattern in MSPSS baseline: one low support, two medium, one high. No pattern in social support described, with two referring to support from family and two saying they did not discuss therapy with family or friends. ***Employment and financial status*** Three of the four severely functionally impaired, with variety in terms of daytime activity (retired, self-employed, not working). ***Capacity to travel to 3MDR clinic*** A combination of driving self to the clinic, but also being in a ‘daze’ on the way home, or public transport. ***The clinic environment*** Strong initial reaction by all four to the clinic, with combinations of anxiety and doubt and also strong impressions of the technology on show.	***Work and social relationships*** Three participants had severe difficulties with work and social relationships at the start of therapy, of whom two improved to a level of significant difficulties and one improved to become sub-clinical. The remaining participant was sub-clinical throughout. ***Depression*** Because of missing data only three cases can be interpreted with regards to depression. All three improved, however they started at different levels of depression (mild, moderate and severe). ***Anxiety*** Three out of the four improved with regards to anxiety, of whom two improved from severe to no anxiety. The fourth remained mildly anxious throughout. ***Social support*** Two out of the four had high levels of perceived social support at the start and end of therapy, whilst one improved from medium to high. One dropped from high to low perceived support. ***Quality of life*** In all quality of life domains there was a mixture of unchanging, improving and worsening scores over the period of the intervention. No patterns in scoring were observed. Perceived health on the day scores were also mixed, with some dramatic changes observed both in terms of improvement and worsening.
Moderate improvement P8 (delayed arm) P10 (delayed arm) P18 (delayed arm) P26 (immediate arm)	***Personality and psychopathology*** All participants living with PTSD, all had previous trauma-focused psychological treatment without loss of PTSD diagnosis. All four had coexisting depression and anxiety. One had extreme PTSD symptoms, one had severe symptoms and two had moderate symptoms. ***Sociodemographic characteristics*** Aged between 45–58. Two educated to degree level or above, one with A level or equivalent, one with school-leaver qualifications. ***Perceived quality of life*** Three of the participants had moderate problems walking about, whilst one had slight problems. Two had no problems washing or dressing themselves, whilst one had slight problems and one had moderate problems. Two had moderate problems undertaking their usual activities, whilst one had severe problems and one had slight problems. Three had moderate pain/discomfort, whilst one had extreme pain/discomfort. Two participants felt that their health on the day was substantially lower than the population average, whilst two scored well below average. ***Trauma type*** All four experienced severe trauma. ***Attitudes and motivations*** Motivated by lack of improvement, interest in new approach and determination. ***Expectations and experiences*** Some apprehension, with therapy experience immersive, intense and exhausting with some worsening of symptoms at first. One dropped out in crisis but still described some good, another contemplated dropping out. ***Cognitive and emotional factors*** Overall, negative valence in choice of phrases was uniformly found to be very predominant in this group. Specifically, anxiety was very predominant. To some extent depression and guilt were also regularly expressed. Phrases with positive valence accounted for approximately 10% of all phrases used. ***Subjective unit of distress*** All four consistently started with extreme levels of distress. Two demonstrated a slight reduction over their sessions but remained extremely distressed. The other two slightly varied over sessions, eventually dropping to severe levels of distress.	***Exposure through walking towards trauma reminders and psychophysiological response*** All four walked very slowly, imposing minimal physical demand. All four had multiple treatment sessions in which they consistently demonstrated a very rapid breathing rate. For three there was a single session where breathing rate was rapid. Three out of the four consistently demonstrated minimally raised heart rates reflective of minimal physical demand. Of these three, one person on occasion demonstrated a heart rate which did not change from their resting rate. The fourth person demonstrated minimally increased heart rates except for one session where heart rate was very high. Only during one session was a very strong heart rate response observed, consistent with what was expected in this context. ***Personal multi-sensory input (selection and sequencing of images and music)*** Finding images a hard task for all four, for a variety of reasons: sourcing pictures, searching during therapy for images able to still elicit strong responses, trauma remaining ‘stuck’ at the end of therapy, and images not eliciting a reaction. ***Working memory task*** All participants engaged in multiple tasks designed to tax working memory, by combining walking, visual tracking of a ball, calling out random numbers and scoring their subjective unit of distress at the end of each image cycle. No specific measures of demand on working memory were available. ***Therapist coaching/relationship*** Important that 3MDR was introduced by trusted therapists, with relationship and consistency important. The participant who dropped out returned to his usual, referring, therapist. ***Number and length of sessions*** Three of the four would have continued with therapy had this been an option, with narratives and issues still to be worked through. The fourth dropped out saying he had ‘too much baggage’. ***Cognitive engagement*** All four had multiple treatment sessions in which they consistently demonstrated a very rapid breathing rate. For three there was a single session where breathing rate was rapid. Very high breathing rates could indicate cognitive engagement. No specific measures of cognitive engagement were available.	***Relationships*, *home environment and social support*** Three of the four reported high support on MSPSS, one medium support. All four supported by family or friends to participate. ***Employment and financial status*** Three of the four severely functionally impaired, one significantly impaired. Variety of daytime activities: volunteering, contract work, one who used 3MDR as opportunity to structure the day, ***Capacity to travel to 3MDR clinic*** Variety of means of transport: mostly driven by wife (once alone), long drive with wife as passenger or long journey via public transport. Not discussed with fourth participant. ***The clinic environment*** Two participants initially struck by technology of the clinic, third likened to being on a ship and fourth had no specific comments.	***Work and social relationships*** Because of missing data only three cases can be interpreted with regards to work and social adjustment. Three participants had severe difficulties with work and social relationships at the start of therapy, of whom two improved to a level of significant difficulties and one remained with severe difficulties. ***Depression*** Because of missing data only three cases can be interpreted with regards to depression. Two improved but started from different levels of depression (severe and moderate). One remained severely depressed throughout. ***Anxiety*** Because of missing data only three cases can be interpreted with regards to anxiety. Three different responses were observed. One improved from severe to moderate anxiety, one remained severely anxious throughout and one worsened from no anxiety to severe. ***Social support*** Because of missing data only three cases can be interpreted with regards to perceived social support. Two had high levels of perceived social support at the start and end of therapy, whilst the third had medium levels of support at the start and the end. ***Quality of life*** Because of missing data only three cases can be interpreted with regards to quality of life. In the mobility domain all scores remained unchanged. In the other quality of life domains there was a mixture of unchanging, improving and worsening scores over the period of the intervention. One participant improved on their perceived health on the day scores, with two starting from substantially below population average and slightly worsening.
Minimal improvement P2 (immediate arm) P25 (immediate arm)	***Personality and psychopathology*** Both participants living with PTSD, both had previous trauma-focused psychological treatment without loss of PTSD diagnosis. Both had coexisting depression and anxiety. One had extreme PTSD symptoms, one had moderate symptoms. ***Sociodemographic characteristics*** Aged between 38–55. One with school-leaver qualifications, one with vocational qualifications. ***Perceived quality of life*** One of the participants had moderate problems walking about, whilst one had slight problems. One had no problems washing or dressing themselves, whilst one had slight problems. Both had moderate problems undertaking their usual activities. One had slight pain/discomfort whilst one had severe pain/discomfort. Both participants felt that their health on the day was well below the population average. ***Trauma type*** Both experienced severe trauma. ***Attitudes and motivations*** Apprehension and fear, motivated by possibility of improvement. One also motivated never to quit. ***Expectations and experiences*** Surprised at the immersive character of therapy, wanting to give 100%. ***Cognitive and emotional factors*** Negative valence in choice of phrases was found to be very predominant for one person in this group, and fractionally predominant for the other. Specifically, anger was predominant closely followed by anxiety, and then depression. Phrases with positive valence accounted for approximately 30% of all phrases used. ***Subjective unit of distress*** Subjective distress varied within the group. One started with extreme levels of distress which gradually dropped to moderate levels at the end. The other started with severe levels of distress which at first dropped to moderate levels before returning to severe at the end.	***Exposure through walking towards trauma reminders and psychophysiological response*** One walked relatively slowly and the other walked very slowly, imposing low to minimal physical demand. The first consistently demonstrated a very rapid breathing rate. The second varied between sessions and demonstrated low, rapid and very rapid breathing responses. Both consistently demonstrated moderately raised heart rates reflective of low physical demand. Stronger heart rate responses were expected in this context. ***Personal multi-sensory input (selection and sequencing of images and music)*** Finding images a hard task for both, with one paying close attention to sequencing during therapy and the second describing finding one particular image as a revelation. ***Working memory task*** Both participants engaged in multiple tasks designed to tax working memory, by combining walking, visual tracking of a ball, calling out random numbers and scoring their subjective unit of distress at the end of each image cycle. No specific measures of demand on working memory were available. ***Therapist coaching/relationship*** Referral to 3MDR therapist by trusted therapist important for both. One knew his 3MDR therapist, which he also saw as helpful. ***Number and length of sessions*** One would have had more sessions only if offered at the outset, one believed session number should be tailored to individual. ***Cognitive engagement*** The first consistently demonstrated a very rapid breathing rate. The second varied between sessions and demonstrated low, rapid and very rapid breathing responses. Very high breathing rates could indicate cognitive engagement. No specific measures of cognitive engagement were available.	***Relationships*, *home environment and social support*** One high support, one medium support. Both talked with either family or friends. ***Employment and financial status*** Both significantly functionally impaired. Daytime activity discussed with one, who adapted working day to accommodate 3MDR. ***Capacity to travel to 3MDR clinic*** Long drive to therapy with loss of concentration reported, with second accompanied by friends. ***The clinic environment*** One familiar with clinic as a gait analysis room, the second found hospital environment challenging.	***Work and social relationships*** Both participants had significant difficulties with work and social relationships at the start of therapy, and both worsened to a level of severe difficulties. ***Depression*** One remained with moderate depression throughout, and one worsened from mild to moderate depression. ***Anxiety*** Both worsened, but started and ended with different levels of anxiety (moderate to severe, and mild to moderate). ***Social support*** Both participants remained the same in terms of social support, one having high levels of perceived social support at the start and end of therapy and the other having medium levels of social at the start and end. ***Quality of life*** In the mobility domain both participants scored worse at the period of the intervention. One scored worse for self-care, whilst one continued to have no problems. For usual activities and pain/discomfort scores for both remained unchanged. Both participants started well below population average for perceived health on the day, and slightly improved over the period of the intervention.

Data summarised in Table 3 above, and presented in greater detail in the S1 File, reveal all 10 participants whose clinical, psychophysiological and qualitative data are synthesised in this paper were extremely, severely or moderately traumatised (assessed using the CAPS-5). All had prior experience of witnessing extreme human suffering and/or death (assessed using the LEC-5). All were recruited into the 3MDR trial because of their continued PTSD symptoms despite having received previous trauma-focused psychological therapies. Beyond this, our exploration of the available data relating to all 10 veterans uncovers patterns between the three therapy response typologies, within the person, intervention and context domains.

With regards to the ‘person’ domain, in contrast to participants in the moderate and minimal improvement groups, dramatic improvers were able to walk without difficulty as measured using baseline ‘mobility’ scores obtained from the E5-5D-5L quality of life tool. Interview data revealed all dramatic improvers as committed and able to complete their courses of therapy, other than one who discontinued early in the context of subjectively experienced transformational benefit. In the moderate improvement group, however, one was unable to continue in the context of psychosocial crisis, one was intensely exhausted by the therapy experience, and one contemplated discontinuing. Three of the four participants in the dramatic improvement group recorded reductions in subjective units of distress, two moving to absent levels by the end of therapy, contrasting with participants in both other groups for whom levels of subjective distress across their courses of therapy were more varied.

Within the ‘intervention’ domain, whilst participants in all response typologies described the task of finding trauma-associated images both practically and psychologically difficult, dramatic improvers included people who were able to source highly charged pictures and to replenish their image selection midway through therapy to continue eliciting a response. Amongst the moderate improvers, in contrast, were participants whose image selections lost their impact over time or who described being ‘stuck’ at the end of their therapy. Of the two minimal improvers, one described difficulty in ordering his images whilst the second described how his selection had centred particularly on the sourcing of one, key, picture. With participants describing the relationship with their treating therapist as important, to be characterised by trust if not also by previous familiarity, the one person who dropped out in a context of psychosocial crisis was part of the moderate improvement group and who returned to their usual therapist. A patterning is also detectable in participants’ expressed interest in having had additional therapy sessions, had that been available as part of the trial protocol. All three moderate improvers who completed therapy would have had additional 3MDR sessions, along with one of the dramatic improvers. Of the two participants in the minimal improvement group, one indicated that he would not have had additional 3MDR sessions unless the number had been agreed at the outset, whilst the other made a case for an individually tailored number of sessions.

Within the ‘context’ domain, all four participants within the dramatic responders group described strong initial reactions to the 3MDR clinic environment, along with two in the moderate responders group. Within the minimally responding group, however, one person was familiar with the technology on show and the other expressed a more generalised concern about the larger hospital environment within which the clinic was located.

Finally, patterns exist within the three response typology groups with regards to the clustering of outcomes, in ways which support our categorisation of participants into response typologies using the trial’s primary outcome measure. In addition to their reductions in PTSD symptomatology as measured using the CAPS-5, outcomes for work and social relationships (using the WASA), depression (using the PHQ-9), anxiety (using the GAD-7) and social support (using the MSPSS) showed a general pattern of improvement (or remained at already-optimal levels) amongst the dramatic responder group. Not all of the participants in the dramatic response group improved, or maintained an optimum, across all secondary outcomes but the patterning of these data particularly contrasted with the two minimal responders. In this group, secondary outcomes suggested a worsening across a number of areas, with moderate responders having a mixed pattern of secondary outcomes across their therapy experiences.

## Discussion

Taking the case of 3MDR, a novel and immersive therapy for people with PTSD, our aim in this paper has been to improve understanding of the interrelationships between people, interventions and context and to explore the interactions between factors within these three domains in different outcome typologies. Following the reporting of principal findings from the main trial from where our data were drawn, which revealed 3MDR as a promising intervention [19], we began by creating the model summarised in Fig 2. We then used this model to frame a four-step convergent approach to the analysis and synthesis of our overall available dataset.

In this context our novel contribution in this new paper is twofold. First is the advancement of our model *per se*, in the anticipation that it has generalisable value in underpinning and structuring detailed, empirical, exploration of the full range of factors associated with the success (or otherwise) of psychological interventions [33, 34]. Second is the use of this model in an exemplifying way to support the granular analysis of factors associated specifically with the provision and receipt of 3MDR, this being a new and novel trauma-focused psychological intervention for people living with PTSD about which relatively little is known of its mechanism of effect [35]. Taken together, our paper advances understanding of what happens when complex interventions are delivered in complex systems to people living with complex difficulties.

Part of the underpinning theory for 3MDR is the idea that the act of walking towards symbolic representations of trauma helps promote engagement and minimise avoidance [17]. Walking *per se* is therefore a crucial part of therapy, and the physical capacity to mobilise for as long as 45–60 minutes during an individual 3MDR session emerges as an important feature in our cross-typology comparison of data within the ‘person’ domain. This is a non-trivial finding, with only the dramatic responder group reporting no mobility difficulties through baseline EQ-5D-5L scores. This has obvious implications for the practical provision of movement-based therapies for people whose capacity to walk is limited. Participants in all three groups described 3MDR as highly immersive, a finding reported in other examinations of 3MDR experiences [36, 37], with the sourcing and use of trauma-associated images commonly described as a challenging task. In this context, our tentative finding that some participants in the moderate and minimal improvement groups experienced difficulties in finding and ordering images able to elicit sustained cognitive and emotional responses, *or* were overwhelmed and exhausted by the high levels of immersion, warrants consideration. Both too much, and too little, exposure and immersion may be associated with less-than-optimal outcomes. We speculate that degrees of engagement and immersion may also be associated with responses to the clinic environment and to the technology of 3MDR. Those who responded to therapy most dramatically were also amongst those initially most impressed by the treatment environment.

Evidence from a recent systematic review suggests that personalised, co-created, virtual reality exposure therapies for veterans with PTSD are beneficial [38]. Evidence also exists that the technology associated, specifically, with 3MDR is acceptable to patients and therapists [39] Consensus work has also commenced to specify the hardware and software necessary for 3MDR [40]. In addition to these ‘technical’ aspects of therapy and the clinic environment as part of the ‘intervention’ domain, our data support the idea that the relationship between therapist and patient remains important. ‘Feeling supported’ is a recurrent theme in van Gelderen et al.’s analysis of treatment processes in, and effects of, 3MDR [41] as is the ‘fireteam partnership’ reported by Hamilton et al. [36]. Trust was important for the participants in our study and the one person (in the moderate responder group) who dropped out whose data are presented here returned immediately to their more familiar therapist. Consistent with the idea that the number of sessions of 3MDR might best be tailored to the individual [36], we also suggest that our finding that the moderate improvers who completed their courses of therapy would all have continued is a potentially important one. Avoiding the feeling of being ‘stuck’, or of ending therapy with some experiences not yet reconsolidated, may demand a flexible approach to 3MDR dosing. This observation is additionally supported by the case of the single dramatic responder who ended therapy early in the context of self-reported, beneficial, transformational change.

We recognise that our identification of patterned data distinguishing aspects of the person, intervention and context domains within the three response typologies sits alongside data where no obvious patterning can be found. Within the person domain, mental health difficulties coexisting with PTSD were common across participants in all three groups, and no clear differences could be discerned in terms of sociodemographic characteristics, experiences of trauma or quality of life (other than mobility, as discussed above). All were motivated by hope that therapy might bring personal relief from trauma and/or hope that others might benefit in the future should 3MDR prove effective. Commonly experienced, across participants in all three response typologies, was an initial apprehension ahead of therapy. The words and phrases uttered by participants in all three groups during their treatment sessions, used in our analysis as a way of comprehending cognitive and emotional factors, had overwhelmingly negative valence. Participants in all three groups were slow walkers, with elevated breathing rate in the absence of a fast pace a generalised finding addressed in a previously published companion paper [30]. We speculate that slow walking pace may have indicated a cautious approach on the part of participants to their personally selected traumatic images, and that very high breathing rates were associated with exposure during sessions. In our model we also consider breathing rate to serve as a proxy for cognitive engagement within the ‘intervention’ domain, but no obvious patterning by response trajectory can be reported. Within the ‘context’ domain we detected no patterning of data between the three groups in terms of social support, or with regards to employment and financial status with people in all typologies experiencing high levels of functional impairment. Within all three groups access to the 3MDR clinic was a significant contextual factor, with reports from those who drove themselves by car including the description of journeys home as a ‘daze’. However, no differences in experiences were identified across typologies.

## Conclusion

With data from only 10 participants available for the analysis reported in this paper care needs to be taken in over-generalising from our interpretations. Our conclusions are suitably cautious, and cannot in and of themselves support confident *a priori* estimations of (a) which groups of people, (b) engaging and responding to therapy in what particular ways, and (c) participating in what particular contexts, are (d) likely to have what types of outcome. However, our analysis does point to some patterning of data reflecting aspects of person, intervention and context across contrasting response typologies. These have value for future therapy provision, focusing attention on the importance of assessing mobility, image selection and use, therapeutic alliance, therapy ‘dosing’ and more.

Our paper may have clearer implications for future research, where our model presented in Fig 2 makes a particular contribution to supporting the search for more personalised interventions. In this paper, the data available to us to exemplify our model in Fig 2 was limited to the best fit available extracted from the already-existing data corpus associated with the larger 3MDR trial. Future research using our model affords *a priori* opportunities to select what types of data should be gathered, thus obviating the need to use proxies (such as breathing rate as a proxy for cognitive engagement, part of the ‘intervention’ domain in our model). The inclusion of measures of therapeutic alliance might also strengthen future studies, as might the generation of qualitative data on participant expectations before actual therapy commences. Subsequent research might also be extended to include the generation of wholly new types of data linked to an expansion of our model. Examples here include additional information on the psychosocial characteristics of participants and physiological responses during therapy sessions, aligned to an expansion of the data collected within the ‘person’ domain.

Finally, we reflect that large numbers of people are known to be living with trauma [42] and that many continue to experience distress despite best-evidence interventions [5]. 3MDR is helpful for some, but it has not been obvious for whom and in what circumstances. Reflecting orientations to intervention development and evaluation which are particularly sensitive to person, content and context [11] our hope is that this paper’s theory-informed synthesis of microscopic, individually relevant, data helps advance the science of a more tailored, personalised, approach to therapy.

## Supporting information

S1 File Data synthesis for 10 participants. (DOCX)

## References

[1] National Institute for Health and Care Excellence. Post-traumatic stress disorder. London: NICE; 2018. 31211536

[2] Kessler RC, Berglund P, Demler O, Jin R, Merikangas KR, Walters EE. Lifetime prevalence and age-of-onset distributions of DSM-IV disorders in the National Comorbidity Survey Replication. Archives of General Psychiatry. 2005;62(6):593–602. doi: 10.1001/archpsyc.62.6.593 15939837

[3] Lewis C, Robert NP, Andrew M, Starling E, Bisson JI. Psychological therapies for post-traumatic stress disorder in adults: systematic review and meta-analysis. European Journal of Psychotraumatology. 2020;11(1):1729633. doi: 10.1080/20008198.2020.1729633 32284821 PMC7144187

[4] Kitchiner N, Lewis C, Roberts NP, Bisson JI. Active duty and ex-serving military personnel with post-traumatic stress disorder treated with psychological therapies: systematic review and meta-analysis. European Journal of Psychotraumatology. 2019;10(1):1684226. doi: 10.1080/20008198.2019.1684226 31762951 PMC6853217

[5] Barawi KS, Lewis C, Simon N, Bisson JI. A systematic review of factors associated with outcome of psychological treatments for post-traumatic stress disorder. European Journal of Psychotraumatology. 2020;11(1):1774240. doi: 10.1080/20008198.2020.1774240 33029317 PMC7473314

[6] Kessler RC, Sonnega A, Bromet E, Hughes M, Nelson CB. Posttraumatic stress disorder in the national comorbidity survey. Archives of General Psychiatry. 1995;52(12):1048–60. doi: 10.1001/archpsyc.1995.03950240066012 7492257

[7] Durham RC, Chambers JA, Power KG, Sharp DM, Macdonald RR, Major KA, et al. Long-term outcome of cognitive behaviour therapy clinical trials in central Scotland. Health Technology Assessment. 2005;9(42):1–174. doi: 10.3310/hta9420 16266559

[8] Foa EB. Prolonged exposure therapy: past, present, and future. Depression and Anxiety. 2011;28(12):1043–7. doi: 10.1002/da.20907 22134957

[9] Duncan C, Weich S, Fenton S-J, Twigg L, Moon G, Madan J, et al. A realist approach to the evaluation of complex mental health interventions. British Journal of Psychiatry. 2018;213(2):451–3. 10.1192/bjp.2018.96 30027875

[10] Moore G, Audrey S, Barker M, Bond L, Bonell C, Hardeman W, et al. Process evaluation of complex interventions. UK Medical Research Council (MRC) guidance. London: MRC Population Health Science Research Network; 2014.

[11] Skivington K, Matthews L, Simpson SA, Craig P, Baird J, Blazeby JM, et al. Framework for the development and evaluation of complex interventions: gap analysis, workshop and consultation-informed update. Health Technology Assessment. 2021;25(57). doi: 10.3310/hta25570 34590577 PMC7614019

[12] Vermetten E, Meijer L, van der Wurff P, Mert A. The effect of military motion-assisted memory desensitization and reprocessing treatment on the symptoms of combat-related post traumatic stress disorder: first preliminary results. In: Wiederhold BK, Riva G, editors. Annual Review of Cybertherapy and Telemedicine 2013. Studies in Health Technology and Informatics. 191. Amsterdam: IOS Press; 2013. p. 125–7. 23792857

[13] Rus-Calafell M, Garety P, Sason E, Craig TJK, Valmaggia LR. Virtual reality in the assessment and treatment of psychosis: a systematic review of its utility, acceptability and effectiveness. Psychological Medicine. 2018;48(3):362–91. doi: 10.1017/S0033291717001945 28735593

[14] Manivannan S, Al-Amri M, Postans M, Westacott LJ, Gray W, Zaben M. The effectiveness of virtual reality interventions for improvement of neurocognitive performance after traumatic brain injury: a systematic review. The Journal of Head Trauma Rehabilitation. 2019;34(2):E52–E65. doi: 10.1097/HTR.0000000000000412 30045223

[15] Lansberg MG, Legault C, MacLellan A, Parikh A, Muccini J, Mlynash M, et al. Home-based virtual reality therapy for hand recovery after stroke. PM&R. 2022;14(3):320–8. doi: 10.1002/pmrj.12598 33773059

[16] Rezaee K, Zolfaghari S. A direct classification approach to recognize stress levels in virtual reality therapy for patients with multiple sclerosis. Computational Intelligence. 2022;38(1):249–68.

[17] van Gelderen M, Nijdam MJ, Vermetten E. An innovative framework for delivering psychotherapy to patients with treatment-resistant posttraumatic stress disorder: rationale for interactive motion-assisted therapy. Frontiers in Psychiatry. 2018;9(176). doi: 10.3389/fpsyt.2018.00176 29780334 PMC5946512

[18] van Gelderen M, Nijdam M, Haagen JFG, Vermetten E. Interactive motion-assisted exposure therapy for veterans with treatment-resistant posttraumatic stress disorder: a randomized controlled trial. Psychotherapy and Psychosomatics. 2020;89(4):215–27. doi: 10.1159/000505977 32203971

[19] Bisson JI, van Deursen R, Hannigan B, Kitchiner N, Barawi K, Jones K, et al. Randomised controlled trial of multi-modular motion-assisted memory desensitisation and reconsolidation (3MDR) for male military veterans with treatment-resistant post-traumatic stress disorder. Acta Psychiatrica Scandinavica. 2020;142(2):141–51. 32495381 10.1111/acps.13200

[20] Jones C, Smith-MacDonald L, Brown MRG, Pike A, Vermetten E, Brémault-Phillips S. Quantitative changes in mental health measures with 3MDR treatment for Canadian military members and veterans. Brain and Behavior. 2022;12(8):e2694. doi: 10.1002/brb3.2694 35849703 PMC9392526

[21] Bisson JI, van Deursen R, Hannigan B, Kitchiner N, Abbott L, Barawi K, et al. Randomised controlled trial of 3MDR for treatment resistant post-traumatic stress disorder (PTSD) in military veterans. Final report to the Forces in Mind Trust 2020. 10.1111/acps.13200 32495381

[22] Weathers FW, Bovin MJ, Lee DJ, Sloan DM, Schnurr PP, Kaloupek DG, et al. The Clinician-Administered PTSD Scale for DSM–5 (CAPS-5): Development and initial psychometric evaluation in military veterans. Psychological Assessment. 2018;30(3):383. doi: 10.1037/pas0000486 28493729 PMC5805662

[23] Weathers FW, Blake DD, Schnurr PP, Kaloupek DG, Marx BP, Keane TM. The life events checklist for DSM-5 (LEC-5). National Center for PTSD; 2013.

[24] Kroenke K, Spitzer RL, Williams JB. The PHQ‐9: validity of a brief depression severity measure. Journal of General Internal Medicine. 2001;16(9):606–13. doi: 10.1046/j.1525-1497.2001.016009606.x 11556941 PMC1495268

[25] Spitzer RL, Kroenke K, Williams JB, Löwe B. A brief measure for assessing generalized anxiety disorder: the GAD-7. Archives of Internal Medicine. 2006;166(10):1092–7. doi: 10.1001/archinte.166.10.1092 16717171

[26] Zimet GD, Dahlem NW, Zimet SG, Farley GK. The Multidimensional Scale of Perceived Social Support. Journal of Personality Assessment. 1988;52(1):30–41. 10.1080/00223891.1990.9674095 2280326

[27] Mundt JC, Marks IM, Shear MK, Greist JM. The Work and Social Adjustment Scale: a simple measure of impairment in functioning. British Journal of Psychiatry. 2002;180(5):461–4. 10.1192/bjp.180.5.461 11983645

[28] Herdman M, Gudex C, Lloyd A, Janssen M, Kind P, Parkin D, et al. Development and preliminary testing of the new five-level version of EQ-5D (EQ-5D-5L). Quality of Life Research. 2011;20(10):1727–36. doi: 10.1007/s11136-011-9903-x 21479777 PMC3220807

[29] Creswell JW, Plano Clark VL. Designing and conducting mixed methods research. 3rd ed. London: Sage; 2018.

[30] van Deursen R, Jones K, Kitchiner N, Hannigan B, Barawi K, Bisson JI. The psychophysiological response during post-traumatic stress disorder treatment with modular motion-assisted memory desensitisation and reconsolidation (3MDR). European Journal of Psychotraumatology. 2021;12(1):1929027. doi: 10.1080/20008198.2021.1929027 34221251 PMC8231381

[31] QSR International Pty Ltd. NVivo qualitative data analysis software (version 12). 12th ed. Burlington, MA: QSR International Pty Ltd; 2018.

[32] Ayres L, Kavanaugh K, Knafl KA. Within-case and across-case approaches to qualitative data analysis. Qualitative Health Research. 2003;13(6):871–83. doi: 10.1177/1049732303013006008 12891720

[33] Palmer L, Thandi G, Norton S, Jones M, Fear NT, Wessely S, et al. Fourteen-year trajectories of posttraumatic stress disorder (PTSD) symptoms in UK military personnel, and associated risk factors. Journal of Psychiatric Research. 2019;109:156–63. doi: 10.1016/j.jpsychires.2018.11.023 30551022

[34] Tarrier N, Sommerfield C, Pilgrim H, Faragher B. Factors associated with outcome of cognitive-behavioural treatment of chronic post-traumatic stress disorder. Behaviour Research and Therapy. 2000;38(2):191–202. doi: 10.1016/s0005-7967(99)00030-3 10661003

[35] Tang E, Jones C, Smith-MacDonald L, Brown MRG, Vermetten E, Brémault-Phillips S. Decreased emotional dysregulation following multi-modal motion-assisted memory desensitization and reconsolidation therapy (3MDR): identifying possible driving factors in remediation of treatment-resistant PTSD. International Journal of Environmental Research and Public Health. 2021;18(22):12243. doi: 10.3390/ijerph182212243 34831999 PMC8621264

[36] Hamilton T, Burback L, Smith-MacDonald L, Jones C, Brown MRG, Mikolas C, et al. Moving toward and through trauma: participant experiences of Multi-Modal Motion-assisted Memory Desensitization and Reconsolidation (3MDR). Frontiers in Psychiatry. 2021;12(779829). doi: 10.3389/fpsyt.2021.779829 35002800 PMC8728006

[37] Jones C, Smith-MacDonald L, Van Veelen N, VanderLaan A, Kaneva Z, Dunleavy RS, et al. Therapist and operator experiences utilizing multi-modal motion-assisted memory desensitization and reconsolidation (3MDR) for treatment of combat related posttraumatic stress disorder amongst military and veteran populations. European Journal of Psychotraumatology. 2022;13(1):2062996. doi: 10.1080/20008198.2022.2062996 35599979 PMC9116239

[38] Vianez A, Marques A, Simões de Almeida R. Virtual reality exposure therapy for armed forces veterans with post-traumatic stress disorder: a systematic review and focus group. International Journal of Environmental Research and Public Health. 2022;19(1):464. doi: 10.3390/ijerph19010464 35010723 PMC8744859

[39] Jones C, Cruz AM, Smith-MacDonald L, Brown MRG, Vermetten E, Brémault-Phillips S. Technology acceptance and usability of a virtual reality intervention for military members and veterans with posttraumatic stress disorder: mixed methods unified theory of acceptance and use of technology study. JMIR Formative Research. 2022;6(4):e33681. 35451971 10.2196/33681 PMC9073607

[40] Jones C, Smith-MacDonald L, Brown MRG, VanDehy J, Grunnet-Jepsen R, Ordek VP, et al. The redesign and validation of multimodal motion-assisted memory desensitization and reconsolidation hardware and software: mixed methods, modified delphi-based validation study. JMIR Human Factors. 2022;9(3):e33682. doi: 10.2196/33682 35819834 PMC9328788

[41] van Gelderen M, Nijdam MJ, Dubbink GE, Sleijpen M, Vermetten E. Perceived treatment processes and effects of interactive motion-assisted exposure therapy for veterans with treatment-resistant posttraumatic stress disorder: a mixed methods study. European Journal of Psychotraumatology. 2020;11(1):1829400. doi: 10.1080/20008198.2020.1829400 33244364 PMC7678674

[42] Karatzias T, Hyland P, Bradley A, Cloitre M, Roberts NP, Bisson JI, et al. Risk factors and comorbidity of ICD‐11 PTSD and complex PTSD: Findings from a trauma‐exposed population based sample of adults in the United Kingdom. Depression and Anxiety. 2019;36(9):887–94. doi: 10.1002/da.22934 31268218

