# Supplementary material for: Factors associated with the outcomes of a novel virtual reality therapy for military veterans with PTSD: Theory development using a mixed methods analysis
Source: PLoS One. 2023 May 25;18(5):e0285763. doi: 10.1371/journal.pone.0285763 (PMC10212105; doi:10.1371/journal.pone.0285763)
Supplement: S1 File — (DOCX) [file pone.0285763.s001.docx]

|  |  | **Supplementary File: Detailed data synthesis for ten participants** | | |  |
| --- | --- | --- | --- | --- | --- |
| **Response typology**  *Based on primary outcome trajectory* | **Participant** | **Person** | **Intervention** | **Context** | **Secondary outcomes**  *Scores reported for period of treatment: baseline to 12 weeks for immediate treatment arm, 12 weeks to 26 weeks for delayed treatment arm* |
| **Dramatic improvement** | P1 (CAPS-5 trajectory during treatment period: 39,13)  Delayed treatment arm | Personality and psychopathology:  Diagnosis: PTSD, with previous trauma-focused psychological treatment without loss of PTSD diagnosis  Comorbidities: PHQ-9 baseline: 4 (no depression), GAD-7 baseline: 4 (no anxiety)  Severity of symptoms and presentation: CAPS-5 baseline: 41 (severe PTSD)  Sociodemographic characteristics:  Age: 66  Education: Apprenticeship  Perceived quality of life:  Baseline scores on the on the EQ-5D-5L:  Mobility: 1 (no problems)  Self-care: 1 (no problems)  Usual activities: 1 (no problems)  Pain/discomfort: 2 (slight problems)  EQ-5D-5L Today: 100 (0=’the worst health you can imagine’, and  100=’the best health you can imagine’)  Trauma type, derived from baseline score on the Life Events Checklist scale (LEC-5):  Experienced natural disasters, combat or exposure to war and serious injury, harm and or death. Witnessed serious injury, harm and death.  Attitudes and motivations:  Jumped at the chance to have 3MDR: ‘anything that can alleviate the symptoms I was getting and make it better to live with, I was up for it’. Also motivated at the prospect of helping others.  Expectations and experiences:  Initially apprehensive.  Found 3MDR immersive: ‘3MDR he described as immersive, saying ‘you’re in a bubble’ and ‘you feel as if you’re going back in time and you’re walking towards it […] I was starting to remember things that I had probably blocked out, there were other things happening, I could remember people who’d been there, people who had turned up’  Cognitive and emotional factors (Displayed words):  Words classed as having overall negative valence were much more frequent than words classed as having overall positive valance, which fluctuated.  Positive valence words gradually increased but still ended much lower than negative valence words.  Anxiety (24%)  Positivity (15%)  Anger (13%)  Depression (12%)  Negativity (11%)  Subjective unit of distress:  SUD was ± 9 (extreme) at the start and gradually dropped to ± 7.5 (severe) at the end. | Exposure through walking towards trauma reminders/exercise and psychophysiological response, derived from recordings of breathing rate, heart rate and walking pace:  BRT: 45-78-75-65-30-45  High response (≥40) at the first and last sessions. Midway very high response (≥60). Moderately high response (≥30) in session 5.  (Normal BRT response to mild exercise is <30).  HRT: 85-70-70-70-65-55  Low response to mild exercise (<90) first time becoming no response subsequently.  Walking speed:  Around 1m/s which is relatively slow for adults  Personal multi-sensory input (selection and sequencing of images and music, prior to and during therapy):  Invested time and effort finding images associated with his major traumas, and was surprised at the detail he found. Finding images was a traumatic experience, his wife describing the detail of the specific events as ‘a terrible thing’. Also devoted time and thought to the selection of two pieces of music, paying attention to lyrics.  Working memory task:  Engaged in multiple tasks designed to tax working memory, by combining walking, visual tracking of a ball, calling out random numbers and scoring subjective unit of distress at the end of each image cycle. No specific measures of demand on working memory were available  SUD was ± 9 (extreme) at the start and gradually dropped to ± 7.5 (severe) at the end.  Therapist coaching/relationship:  Therapist put him at ease.  Number and length of sessions:  Did not consider stopping the therapy: ‘Anything was better than just carrying on the way we were’. Knowing that other people had gone to a lot of bother was also important, combined with a sense of commitment to see things through. Would have had additional therapy if available.  Cognitive engagement, derived from recordings of breathing rate, as a proxy for more direct measures of cognitive engagement:  BRT: 45-78-75-65-30-45  High response (≥40) at the first and last sessions. Midway very high response (≥60). Moderate response (≥30) in session 5.  (Normal BRT response to mild exercise is <30). | Relationships, home environment and social support:  Baseline score on the Multidimensional Scale for Perceived Social Support scale (MSPSS): 12 (low support)  Supported by his wife, who he said described him as being less aggressive, and calmer, at the end.  Employment and financial status:  Baseline score on the Work and Social Adjustment scale (WSAS): 8 (sub-clinical functional impairment)  Retired.  Capacity to travel to 3MDR clinic:  Travelled by public transport, a routine made relatively easy now he no longer worked.  The clinic environment:  ‘I was very impressed when I got in the room, it was like NASA’. He also felt put at ease during this first clinic visit, and felt ‘this is ok, I can cope with this’ as everything was explained. Even during his first try-out on the treadmill (without his selected images) he felt anxious, however: ‘oh gosh I shouldn’t have done this, I shouldn’t have come because you felt, you felt closed in, you were into that, pushed into that tunnel’. | Changes recorded over time in scores on the 3MDR trial’s secondary outcome measures  WSAS Trajectory: 7,1 (sub-clinical to sub-clinical difficulties with work, social activities and relationships)  PHQ-9 Trajectory: 7,3 (mild to no depression)  GAD-7 Trajectory: 8,7 (mild to mild anxiety)  MSPSS Trajectory: 84,84 (high to high social support)  EQ-5D-5L Trajectory:  Mobility: 1,1 (no problems to no problems)  Self-care: 1,1 (no problems to no problems)  Usual activities: 3,1 (moderate problems to no problems)  Pain/discomfort: 1,1 (no problems to no problems)  EQ-5D-5L Today Trajectory:  90,95  (0=’the worst health you can imagine’, and 100=’the best health you can imagine’) |
|  | P4 (CAPS-5 trajectory during treatment period: 38, 14)  Immediate treatment arm | Personality and psychopathology:  Diagnosis: PTSD, with previous trauma-focused psychological treatment without loss of PTSD diagnosis  Comorbidities: PHQ-9 baseline: 13 (moderate depression), GAD-7 baseline: 13 (moderate anxiety)  Severity of symptoms and presentation: CAPS-5 baseline: 38 (severe PTSD)  Sociodemographic characteristics:  Age: 49  Education: 5+GCSEs or equivalent  Perceived quality of life:  Baseline scores on the on the EQ-5D-5L:  Mobility: 1 (no problems)  Self-care: 1 (no problems)  Usual activities: 1 (no problems)  Pain/discomfort: 3 (moderate problems)  EQ-5D-5L Today: 70  (0=’the worst health you can imagine’, and  100=’the best health you can imagine’)  Trauma type, derived from baseline scores on the Life Events Checklist scale (LEC-5):  Experienced transportation accident, physical assault, sexual assault, exposure to combat or war and childhood physical assault. Witnessed fire explosion and severe human suffering.  33 years since trauma experiences  Attitudes and motivations:  Open to 3MDR because of what it might do for others: ‘if it don’t help me it might help someone else and you needed participants so I agreed to give it a go’  Expectations and experiences:  Found information online, and described experience thus: ‘I didn’t know what to expect it was just it was quite overwhelming the whole 3-D experience on the treadmill because you do get sucked into it when you’re there and the screen is virtually surrounding you, yeah you sort of get sucked in to the, you’re in that picture’  Cognitive and emotional factors (Displayed words):  Words classed as having overall negative valence were much more frequent than words classed as having positive valance, and these dropped and then rose again. Positive valence words increased substantially and were more frequent than negative midway but dropped again and ended much lower than negative valence words  Positivity (25%)  Anxiety (18%)  Anger (16%)  NOS (15%)  Negativity (14%)  Subjective unit of distress:  SUD was ± 3.5 (mild) at the start and gradually increased to above 5 (moderate) at the end. | Exposure through walking towards trauma reminders/exercise and psychophysiological response, derived from recordings of breathing rate, heart rate and walking pace:  BRT: 40-**90**-55-35-40-**65**  Mixed responses with very high response second and last sessions. In between mostly high responses and a moderately high response. The second session had a particularly high response  (Normal BRT response to mild exercise is <30)  HRT: 100-110-105-105-110-90  Normal response (90-110) to mild walking exercise  Walking speed:  Around 1-1.5m/s which is normal range for adults  Personal multi-sensory input (selection and sequencing of images and music, prior to and during therapy):  He searched, on his own, for images (some being ‘rather random, others quite gruesome’) and brought a bank of 12-15 to his therapist which were cut down to around seven, but over the course of therapy were replenished via fresh searching. His music choice reflected his service, associated with a tv programme, which he described as ‘poignant’ and which was followed by music which he changed over the course of therapy. Images moved out and in over sessions, reflecting their effect.  Working memory task:  Engaged in multiple tasks designed to tax working memory, by combining walking, visual tracking of a ball, calling out random numbers and scoring subjective unit of distress at the end of each image cycle. No specific measures of demand on working memory were available  SUD was ± 3.5 (mild) at the start and gradually increased to above 5 (moderate) at the end  Therapist coaching/relationship:  Coaching: trusted therapist who was familiar  Number and length of sessions:  Would not have dropped out: ‘No, once I commit to something I’m pretty committed to it. It would have had to have been something really weird or strange for me to drop out.’  Looking back, he believed 3MDR ‘has done a lot of good’ but also that ‘only time can tell’  Cognitive engagement  Derived from recordings of breathing rate, as a proxy for more direct measures of cognitive engagement:  BRT: 40-**90**-55-35-40-**65**  Mixed responses with very high response second and last sessions. In between mostly high responses and a moderately high response. The second session had a particularly high response  (Normal BRT response to mild exercise is <30) | Relationships, home environment and social support:  Baseline score on the Multidimensional Scale for Perceived Social Support scale (MSPSS): 55 (high support)  Did not speak with family or friends between visits.  Employment and financial status:  Baseline score on the Work and Social Adjustment scale (WSAS): 27 (severe functional impairment)  Self-employed, so scheduling clinic visits not a problem but did lose a day’s pay each visit.  Capacity to travel to 3MDR clinic:  Drove to and from the clinic, but sometimes felt in a ‘daze’ and so would wait before driving home.  The clinic environment:  His reaction to first seeing the clinic was, ‘This is different’. | Changes recorded over time in scores on the 3MDR trial’s secondary outcome measures  WSAS Trajectory: 27,11 (severe to significant difficulties with work, social activities and relationships)  PHQ-9 Trajectory: 13,8 (moderate to mild depression)  GAD-7 Trajectory: 13,6 (moderate to mild anxiety)  MSPSS Trajectory: 55,56 (high to high social support)  EQ-5D-5L Trajectory:  Mobility: 1,3 (no problems to moderate problems)  Self-care: 1,3 (no problems to moderate problems)  Usual activities: 1,2 (no problems to slight problems)  Pain/discomfort: 3,4 (moderate problems to severe problems)  EQ-5D-5L Today Trajectory:  70,35  (0=’the worst health you can imagine’, and 100=’the best health you can imagine’) |
|  | P9 (CAPS-5 trajectory during treatment period: 38, 0)  Immediate treatment arm | Personality and psychopathology:  Diagnosis: PTSD, with previous trauma-focused psychological treatment without loss of PTSD diagnosis  Comorbidities: PHQ-9 baseline: 19 (moderately severe depression), GAD-7 baseline: 16 (severe anxiety)  Severity of symptoms and presentation: CAPS-5 baseline: 38 (severe PTSD)  Sociodemographic characteristics:  Age: 42  Education: 1-4 GCSEs or equivalent  Perceived quality of life:  Baseline scores on the on the EQ-5D-5L:  Mobility: 1 (no problems)  Self-care: 1 (no problems)  Usual activities: 3 (moderate problems)  Pain/discomfort: 1 (no problems)  EQ-5D-5L Today: 65  (0=’the worst health you can imagine’, and  100=’the best health you can imagine’)  Trauma type, derived from baseline scores on the Life Events Checklist scale (LEC-5):  Experienced physical assault, combat or exposure to war, serious injury or harm and childhood physical abuse. Witnessed sudden violent death.  Attitudes and motivations:  When offered the chance to have 3MDR ‘I jumped feet first in’, saying ‘I’d have experienced anything that was given me to make me feel better’.  Expectations and experiences:  His preference with his images was to start with the most loaded, and work down: ‘I chose mostly the hard ones, because, like I say I wanted to jump in feet first, I didn’t want to start with like a two or a three, I wanted to start with like a 10 then a nine and an eight’. The process of walking he described as important: ‘as you’re walking I’d think, ‘I can do this this’, you’re walking into the picture, as the picture is coming towards you, and it is significant to therapy because you’re taking your mind off things as well’. Would definitely recommend 3MDR to others: ‘This has been the best therapy that I’ve ever responded to, in my eyes. This has done wonders to me. Wonders’, with a resolution of nightmares, and no more drinking.  Cognitive and emotional factors (Displayed words):  Words classed as having overall negative valence were much more frequent than words classed as having overall positive valence at first, substantially dropping to end up quite low. Positive valence words gradually increased and ended higher than negative valence words.  Negativity (24%)  Depression (21%)  Anger (15%)  Guilt (14%)  Positivity (12%)  Note:  Anxiety only 2%  Subjective unit of distress:  SUD was ± 8.5 (extreme) at the start and rapidly dropped to ± 1 (absent) at the end of 4 sessions. | Exposure through walking towards trauma reminders/exercise and psychophysiological response, derived from recordings of breathing rate, heart rate and walking pace:  BRT: 20-20-25-45  Started with normal response (<30) but last session high (≥40)  (Normal BRT response to mild exercise is <30)  HRT: **125**-xx-105-**120**  First and last session moderate response to mild exercise with normal response between  Walking speed:  Around 1m/s which is relatively slow for adults  Personal multi-sensory input (selection and sequencing of images and music, prior to and during therapy):  His images ‘were very distressing for me at the beginning’, and involved internet searching in relation to a specific trauma: ‘I didn’t want to look for those pictures and specific points at that time because, well, that’s the reason why I done the study, because of that key specific time’. His then-partner helped, but ‘each one brought tears to my eyes, and it was just horrible, to look for it, but as you see them from time to time they become more easy to look at’. Selecting a song associated with his trauma was also hard, ‘because it made, it was like how I felt at that time’ though the second track was much easier to select  Working memory task:  Engaged in multiple tasks designed to tax working memory, by combining walking, visual tracking of a ball, calling out random numbers and scoring subjective unit of distress at the end of each image cycle. No specific measures of demand on working memory were available  SUD was ± 8.5 (extreme) at the start and rapidly dropped to ± 1 (absent) at the end of 4 sessions  Therapist coaching/relationship:  Although he did not know his therapist until starting in the trial he had trust in him (‘he’s an outstanding bloke’)  Number and length of sessions:  His decision to end early was because ‘when the photos came up I wasn’t so stressed, I wasn’t so blubbery, I was different’  Cognitive engagement  Derived from recordings of breathing rate, as a proxy for more direct measures of cognitive engagement:  BRT: 20-20-25-45  Started with normal response (<30) but last session high (≥40).  (Normal BRT response to mild exercise is <30) | Relationships, home environment and social support  Baseline score on the Multidimensional Scale for Perceived Social Support scale (MSPSS): 52 (medium support)  Spoke to partner and mother about therapy experiences, and with other veterans  Employment and financial status  Baseline score on the Work and Social Adjustment scale  (WSAS): 35 (severe functional impairment)  Was off work during therapy so scheduling was not a problem.  Capacity to travel to 3MDR clinic:  Drove to and from the clinic  The clinic environment:  Of his first visit to the clinic he said: ‘It was like walking into a, what I would, what I would call a spaceship, because when you walk into the room you’ve got those cameras everywhere, even though they don’t, they’re not for the actual therapy itself | Changes recorded over time in scores on the 3MDR trial’s secondary outcome measures  WSAS Trajectory: 35,15 (severe to significant difficulties with work, social activities and relationships )  PHQ-9 Trajectory: 19,99 (moderately severe depression then missing data)  GAD-7 Trajectory: 16,0 (severe to no anxiety)  MSPSS Trajectory: 52, 84 (medium to high social support)  EQ-5D-5L Trajectory:  Mobility: 1,1 (no problems to no problems)  Self-care: 1,1 (no problems to no problems)  Usual activities: 3,1 (moderate problems to no problems)  Pain/discomfort: 1,1 (no problems to no problems)  EQ-5D-5L Today Trajectory: 65,85  (0=’the worst health you can imagine’, and 100=’the best health you can imagine’) |
|  | P12 (CAPS-5 trajectory during treatment period: 39,4)  Delayed treatment arm | Personality and psychopathology:  Diagnosis: PTSD, with previous trauma-focused psychological treatment without loss of PTSD diagnosis  Comorbidities: PHQ-9 baseline: 20 (severe depression), GAD-7 baseline: 16 (severe anxiety)  Severity of symptoms and presentation: CAPS-5 baseline: 55 (extreme PTSD)  Sociodemographic characteristics:  Age:55  Education: Degree level or above  Perceived quality of life:  Baseline scores on the on the EQ-5D-5L:  Mobility: 1 (no problems)  Self-care: 1 (no problems)  Usual activities: 3 (moderate problems)  Pain/discomfort: 4 (severe problems)  EQ-5D-5L Today: 30  (0=’the worst health you can imagine’, and  100=’the best health you can imagine’)  Trauma type, derived from baseline scores on the Life Events Checklist scale (LEC-5):  Experienced fire or explosion, transport accident, serious accident at work, physical assault, assault with a weapon, combat or exposure to war, life threatening illness, sudden violent death, sudden unexpected death someone close to you, and serious injury. Witnessed natural disaster, transport accident, physical assault, combat or exposure to war, sudden violent death, and serious injury.  Attitudes and motivations:  Having slipped back was open to new therapy, as says he is ‘interested in new things’.  Expectations and experiences:  Therapy itself he found helpful, and described swapping photos in and out over sessions and that during 3MDR ‘the penny dropped’, during sessions 2 and 3 specifically. Coming off the treadmill he said how he had ‘never moved on’, but that walking towards his images was ‘like being in the real thing’. He also described ‘a blip’, where in between sessions he ‘was on a bit of a downer’ and thought he was slipping back.  Cognitive and emotional factors (Displayed words):  Words classed as having overall negative valence were much more frequent than words classed as having overall positive valence at first, and rapidly reduced. Positive valence words were minimal at first and gradually increased ending just lower than negative valence words  Anxiety (22%)  Negativity (22%)  Positivity (14%)  Depression (10%)  Physical NOS (10%)  Subjective unit of distress:  SUD was under 8 (severe) at the start and rapidly dropped to under 1 (absent) at the end. | Exposure through walking towards trauma reminders/exercise and psychophysiological response, derived from recordings of breathing rate, heart rate and walking pace:  BRT: 12-50-**60**-35-35-35  After no response first time, session 2 high and session 3 very high response. Last 3 sessions moderately high response.  (Normal BRT response to mild exercise is <30).  HRT: 90-105-105-95-105-70  Normal response to mild exercise with last session a low response.  Walking speed:  Around 1m/s which is relatively slow for adults. First session was extra slow.  Personal multi-sensory input (selection and sequencing of images and music, prior to and during therapy):  He began searching for images a week before, looking for ‘photographs that did something’. This was a hard task, as he ‘wanted ones that really upset me, like I don’t know why just you know, there was a point, I thought it was pointless going there with photographs that weren’t going to do anything’. Bringing his selection to his therapist led to some being dropped because ‘There was a couple, that weren’t too good and didn’t sort of provoke any thought’. Music choices were easily made, in contrast.  Therapy itself he found helpful, and described swapping photos in and out over sessions and that during 3MDR ‘the penny dropped’, during sessions 2 and 3 specifically  Working memory task:  Engaged in multiple tasks designed to tax working memory, by combining walking, visual tracking of a ball, calling out random numbers and scoring subjective unit of distress at the end of each image cycle. No specific measures of demand on working memory were available  SUD was under 8 (severe) at the start and rapidly dropped to under 1 (absent) at the end  Therapist coaching/relationship:  3MDR provided by familiar therapist  Number and length of sessions:  He would not have continued with therapy had it been available, describing himself as now being ‘a better person […] happier’  Cognitive engagement, derived from recordings of breathing rate, as a proxy for more direct measures of cognitive engagement:  BRT: 12-50-**60**-35-35-35  After no response first time, session 2 high and session 3 very high response. Last 3 sessions moderately high response  (Normal BRT response to mild exercise is <30) | Relationships, home environment and social support:  Baseline score on the Multidimensional Scale for Perceived Social Support scale (MSPSS): 42 (medium support)  He made the decision to join the study without consulting family or friends, saying how ‘My wife doesn’t get involved with anything like that’  Employment and financial status:  Baseline score on the Work and Social Adjustment scale (WSAS): 28 (severe functional impairment)  Talking about accommodating 3MDR into regular schedule said: ‘I don’t do a lot, it was quite easy actually you know, I just, my life was quite boring’  Capacity to travel to 3MDR clinic:  Scheduling therapy he described as straightforward, and involved driving to and from the clinic  The clinic environment:  His initial thoughts were that he ‘was quite wary’, and on first visiting the clinic and seeing the set-up he said how ‘I didn’t think it was going to work, you know, I had some doubt but I’m quite good at entering a thing with an open mind’ | Changes recorded over time in scores on the 3MDR trial’s secondary outcome measures  WSAS Trajectory: 33,4 (severe to subclinical difficulties with work, social activities and relationships)  PHQ-9 Trajectory: 26,9 (severe to mild depression)  GAD-7 Trajectory: 20,3 (severe to no anxiety)  MSPSS Trajectory: 54,12 (high to low social support)  EQ-5D-5L Trajectory:  Mobility: 2,3 (slight problems to moderate problems)  Self-care: 3,2 (moderate problems to slight problems)  Usual activities: 4,3 (severe problems to moderate problems)  Pain/discomfort: 4,4 (severe problems to severe problems)  EQ-5D-5L Today Trajectory: 15,75  (0=’the worst health you can imagine’, and 100=’the best health you can imagine’) |
| **Moderate improvement** | P8 (CAPS-5 trajectory during treatment period: 32,13)  Delayed treatment arm | Personality and psychopathology:  Diagnosis: PTSD, with previous trauma-focused psychological treatment without loss of PTSD diagnosis  Comorbidities: PHQ-9 baseline: 24 (severe depression), GAD-7 baseline: 20 (severe anxiety)  Severity of symptoms and presentation: CAPS-5 baseline: 51 (extreme PTSD)  Sociodemographic characteristics:  Age: 58  Education: A level or equivalent  Perceived quality of life:  Baseline scores on the on the EQ-5D-5L:  Mobility: 2 (slight problems)  Self-care: 2 (slight problems)  Usual activities: 4 (severe problems)  Pain/discomfort: 5 (extreme problems)  EQ-5D-5L Today: 30  (0=’the worst health you can imagine’, and  100=’the best health you can imagine’)  Trauma type, derived from baseline scores on the Life Events Checklist scale (LEC-5):  Experienced serious accident at work, exposure to toxic substance, physical assault, sexual assault, other unwanted sexual assault, combat or exposure to war, life threatening illness, serious injury harm or death, any other stressful event. Witnessed natural disaster, assault with a weapon, severe human suffering, sudden violent death and sudden unexpected death of someone close to you  Attitudes and motivations:  Having served in multiple conflicts and experienced multiple traumas said that ‘anything is better than nothing’.  Expectations and experiences:  Described being ‘apprehensive’ ahead of commencing. His first three or four therapy sessions saw a worsening of his difficulties, characterised by nightmares, feeling drained and ‘reliving what I’d gone through’  Cognitive and emotional factors (Displayed words):  Words classed as having overall negative valence were much more frequent than words classed as having overall positive valence at first, substantially dipping midway and then rising again. Positive valence words gradually increased but still ended much lower than negative valence words  Anxiety (21%)  Guilt (18%)  Depression (14%)  Positivity (12%)  Negativity (11%)  Subjective unit of distress:  SUD was 10 (extreme) at the start and finally dropped to ± 9 (extreme) for the last two sessions | Exposure through walking towards trauma reminders/exercise and psychophysiological response, derived from recordings of breathing rate, heart rate and walking pace:  BRT: 45-45-55-30-40-40  All high responses except session 4 with moderate response  (Normal BRT response to mild exercise is <30).  HRT: 90-90-95-85-100-100  Normal responses to mild exercise  Walking speed:  Around <1m/s which is slow for adults  Personal multi-sensory input (selection and sequencing of images and music, prior to and during therapy):  Some of his images were his own, though many had disappeared over the years, and others were sourced online and were chosen to reflect his long service. He had had no cause to look at service-related photos for many years, and finding them evoked strong memories (excitement at going into conflict, guilt at seeing images of missiles being fired at civilians, warships). His music choices were meaningful, including a song associated with a return home from conflict    Working memory task:  Engaged in multiple tasks designed to tax working memory, by combining walking, visual tracking of a ball, calling out random numbers and scoring subjective unit of distress at the end of each image cycle. No specific measures of demand on working memory were available  SUD was 10 (extreme) at the start and finally dropped to ± 9 (extreme) for the last two sessions  Therapist coaching/relationship:  The introduction to 3MDR was made by his existing therapist, who delivered the therapy, and who helped explain trickier terms in the information sheet  Number and length of sessions:  Would have accepted more therapy sessions had they been available, with wife saying how he still has ‘issues’  Cognitive engagement  Derived from recordings of breathing rate, as a proxy for more direct measures of cognitive engagement:  BRT: 45-45-55-30-40-40  All high responses except session 4 with moderate response  (Normal BRT response to mild exercise is <30) | Relationships, home environment and social support:  Baseline score on the Multidimensional Scale for Perceived Social Support scale (MSPSS): 76 (high support)  Strongly encouraged by his wife (present at the interview) to join the study (‘I’m always encouraging him’)  Employment and financial status:  Baseline score on the Work and Social Adjustment scale (WSAS): 28 (severe functional impairment)  Weekly schedule involved voluntary work, and 3MDR meant having to change some existing commitments.  Capacity to travel to 3MDR clinic:  His wife drove him for most sessions, after he drove himself home on one occasion saying how ‘I didn’t know how I got home, that’s being totally honest, I did not know […].’  The clinic environment:  He likened his first clinic visit to being on a ship, paying particular attention to the pipes in the room as opposed to the actual equipment. | Changes recorded over time in scores on the 3MDR trial’s secondary outcome measures  WSAS Trajectory: 24,12 (severe to significant difficulties with work, social activities and relationships)  PHQ-9 Trajectory: 24,17 (severe to moderately severe depression)  GAD-7 Trajectory: 3,19 (no to severe anxiety)  MSPSS Trajectory: 79,80 (high to high social support)  EQ-5D-5L Trajectory:  Mobility: 3,3 (moderate problems to moderate problems)  Self-care: 3,2 (moderate problems to slight problems)  Usual activities: 3,4 (moderate problems to severe problems)  Pain/discomfort: 4,5 (severe problems to extreme problems)  EQ-5D-5L Today Trajectory: 45,40  (0=’the worst health you can imagine’, and 100=’the best health you can imagine’) |
|  | P10 (CAPS-5 trajectory during treatment period: 30,11)  Delayed treatment arm | Personality and psychopathology:  Diagnosis: PTSD, with previous trauma-focused psychological treatment without loss of PTSD diagnosis  Comorbidities: PHQ-9 baseline: 11 (moderate depression), GAD-7 baseline: 11 (moderate anxiety)  Severity of symptoms and presentation: CAPS-5 baseline: 52 (extreme PTSD)  Sociodemographic characteristics:  Age: 45  Education: Degree level or above  Perceived quality of life:  Baseline scores on the on the EQ-5D-5L:  Mobility: 3 (moderate problems)  Self-care: 1 (no problems)  Usual activities: 2 (slight problems)  Pain/discomfort: 3 (moderate problems)  EQ-5D-5L Today: 55  (0=’the worst health you can imagine’, and  100=’the best health you can imagine’)  Trauma type, derived from baseline scores on the Life Events Checklist scale (LEC-5):  Experienced fire or explosion, assault with a weapon, combat or exposure to war, and serious injury or harm. Witnessed serious accident at work, physical assault, combat or exposure to war, severe human suffering, sudden violent death to someone and unexpected death of someone close to you.  Attitudes and motivations:  Keen to join the study, saying his illness ‘has sort of plateaued’ after 5-6 years of treatment.  Expectations and experiences:  Chose not to over-research 3MDR before starting, because ‘I wanted to come in with a complete open mind on the whole thing’, but was also fearful ‘because I knew they’d be unlocking a lot of bad things inside me’.  Therapy continued to be deeply affecting, and images triggered an ‘horrendous reaction’. Walking was slow (‘my knees are knackered’), and whilst session 1 was difficult the experience intensified over time: ‘the first, probably about halfway through and I started to feel exhausted and up to the end of it feeling almost like you’re feeling a collapse, the second and some of the sessions after that were worse than that but I was feeling utterly exhausted on that first session but the second one got a lot worse from there on’. The music, the images, the talk all combined: ‘I never felt like this before in my life’.  ‘It’s definitely helped me without a shadow of a doubt, more than other therapy I’ve had, it’s been the hardest therapy but it has definitely helped me’.  Cognitive and emotional factors (Displayed words):  Words classed as having overall negative valence were much more frequent than words classed as having overall positive valence at first, before gradually reducing by a small amount. Positive valence words were non-existent at first but in the last two sessions increased quite rapidly but ended lower than negative valence words  Anxiety (45%)  Negativity (14%)  Depression (8%)  Positivity (7%)  Guilt (7%)  Subjective unit of distress:  Missing data session 1  SUD was above 8 (extreme) at the start increased to above 9 and dropped again to under 7 (severe) at the end | Exposure through walking towards trauma reminders/exercise and psychophysiological response, derived from recordings of breathing rate, heart rate and walking pace:  BRT: 50-40-35-50-55-**70**  At start high response. Midway moderately high response becoming high again and at end very high response  (Normal BRT response to mild exercise is <30).  HRT: 85-80-55-95-85-65  Mixed response to mild exercise mostly low and one normal response  Walking speed:  Under 1m/s which is slow for adults  Personal multi-sensory input (selection and sequencing of images and music, prior to and during therapy):  He searched on his own for images, selecting photos relating to two areas (dealing with dead people, and a suicide attack). Over time his response to images changed, and he was determined that he was ‘not going to leave any stone unturned’. His music choices included music which would ‘get all the crying out of me, so I won’t come in through the door crying’  An important consideration was the selection of images, in sessions, to tell a story in sequence as opposed to images being displayed from disconnected events  Working memory task:  Engaged in multiple tasks designed to tax working memory, by combining walking, visual tracking of a ball, calling out random numbers and scoring subjective unit of distress at the end of each image cycle. No specific measures of demand on working memory were available  Missing data session 1.  SUD was above 8 (extreme) at the start increased to above 9 and dropped again to under 7 (severe) at the end  Therapist coaching/relationship:  Having a consistent therapist across sessions was important, and this therapist he knew and trusted  Number and length of sessions:  He would have continued with therapy, having more narratives to work through  Cognitive engagement  Derived from recordings of breathing rate, as a proxy for more direct measures of cognitive engagement:  BRT: 50-40-35-50-55-**70**  At start high response. Midway moderately high response becoming high again and at end very high response  (Normal BRT response to mild exercise is <30) | Relationships, home environment and social support:  Baseline score on the Multidimensional Scale for Perceived Social Support scale (MSPSS): 72 (high support)  Encouraged by his wife to have 3MDR, ‘because she’s living with someone who’s mentally ill’, but had no close friends to also talk with  Employment and financial status:  Baseline score on the Work and Social Adjustment scale (WSAS): 16 (significant functional impairment)  His work was on a contract basis, and the company he worked for knew he had been ill and were able and willing to make adjustments  Capacity to travel to 3MDR clinic:  Not discussed during interview  The clinic environment:  He arrived in tears to his first clinic visit, describing himself as ‘vulnerable’. He had good explanations of the set-up from his therapist and an RA, but said: ‘it reminds me of 28 Days Later and fucking chimpanzees tied to a thing and you think bloody hell you know, an experiment at Porton Down’ | Changes recorded over time in scores on the 3MDR trial’s secondary outcome measures  WSAS Trajectory: 21,14 (severe to significant difficulties with work, social activities and relationships)  PHQ-9 Trajectory: 17,7 (moderately severe to mild depression)  GAD-7 Trajectory: 15,13 (severe to moderate anxiety)  MSPSS Trajectory: 66,61 (high to high social support)  EQ-5D-5L Trajectory:  Mobility: 3,3 (moderate problems to moderate problems)  Self-care: 1,1 (no problems to no problems)  Usual activities: 2,1 (slight problems to no problems)  Pain/discomfort: 2,2 (slight problems to slight problems)  EQ-5D-5L Today Trajectory:  40,75  (0=’the worst health you can imagine’, and 100=’the best health you can imagine’) |
|  | P18 (CAPS-5 trajectory during treatment period: 38,18)  Delayed treatment arm | Personality and psychopathology:  Diagnosis: PTSD, with previous trauma-focused psychological treatment without loss of PTSD diagnosis  Comorbidities: PHQ-9 baseline: 23 (severe depression), GAD-7 baseline: 19 (severe anxiety)  Severity of symptoms and presentation: CAPS-5 baseline: 41 (severe PTSD)  Sociodemographic characteristics:  Age: 53  Education: 1-4 GCSEs  Perceived quality of life:  Baseline scores on the on the EQ-5D-5L:  Mobility: 3 (moderate problems)  Self-care: 3 (moderate problems)  Usual activities: 3 (moderate problems)  Pain/discomfort: 3 (moderate problems)  EQ-5D-5L Today: 60  (0=’the worst health you can imagine’, and  100=’the best health you can imagine’)  Trauma type, derived from baseline scores on the Life Events Checklist scale (LEC-5):  Experienced fire or explosion, transportation accident, serious accident at work, exposure to toxic substances, physical assault, assault with a weapon, combat or exposure to war, life threatening illness, sudden unexpected death you caused. Witnessed fire or explosion, physical assault, and severe human suffering. Not sure about serious injury harm you caused  Attitudes and motivations:  Having heard of the trial from his familiar, trusted referring therapist, he was keen to take part in the study saying how ‘you’re on a treadmill and you’re walking through a war zone and I thought you know, instantly I thought wow, that’s, for me personally’  Expectations and experiences:  Taking part in the study was physically and mentally draining, and he thought of dropping out following one very difficult session: ‘I think it was about halfway through or just after and I was pretty close thinking mmm, because I think it was just that particular day that you know, felt like a kick in the teeth like and because I felt so shit and drained and all the rest of it I just thought how much more, am I going to put up with this […]  Cognitive and emotional factors (Displayed words):  Words classed as having overall negative valence were much more frequent than words classed as having overall positive valence at first, fluctuating to end somewhat reduced Positive valence words were minimal at first but gradually increased but ended lower than negative valence words  Anxiety (25%)  Negativity (18%)  Anger (14%)  Positivity (13%)  Depression (11%)  Subjective unit of distress:  SUD was above 8 (extreme) at the start fluctuated to above 7 (severe) at the end | Exposure through walking towards trauma reminders/exercise and psychophysiological response, derived from recordings of breathing rate, heart rate and walking pace:  BRT: **65**-30-50-**80**-55-50  First very high response, session 2 moderate, session 3 high, session 4 very high, final 2 are high responses  (Normal BRT response to mild exercise is <30)  HRT: 100-**170**-100-90-90-90  Normal responses to mild exercise except for session 2 where the HR is very high  Walking speed:  Around 0.7m/s which is very slow for adults  Personal multi-sensory input (selection and sequencing of images and music, prior to and during therapy):  Searching for music was straightforward, but more demanding was locating images with the majority being sourced via the internet. One trauma related to losing friends, which he described as ‘my little box stuck at the back of my head’, which he described as still being ‘stuck’ at the end of his therapy  Images were generally selected at the start of each session, with the most emotionally charged tending to be used at the end of the session modified by some random shuffling, in some sessions, of the ordering of the selected pictures  Working memory task:  Engaged in multiple tasks designed to tax working memory, by combining walking, visual tracking of a ball, calling out random numbers and scoring subjective unit of distress at the end of each image cycle. No specific measures of demand on working memory were available  SUD was above 8 (extreme) at the start fluctuated to above 7 (severe) at the end  Therapist coaching/relationship:  Referred by trusted therapist, and built a relationship with treating therapist: ‘she would let me talk how I’d feel afterwards or halfway through or whatever and she could see how I was you know, and emotional’  Number and length of sessions:  At the end of the therapy he would have continued had the option been available: ‘if it was like what I’ve just done is the first part and then you got a second part, then yeah I think the challenge would be still there’  Cognitive engagement  Derived from recordings of breathing rate, as a proxy for more direct measures of cognitive engagement:  BRT: **65**-30-50-**80**-55-50  First very high response, session 2 moderate, session 3 high, session 4 very high, final 2 are high responses  (Normal BRT response to mild exercise is <30) | Relationships, home environment and social support:  Baseline score on the Multidimensional Scale for Perceived Social Support scale (MSPSS): 60 (high support)  Determination, and a sense of having been fortunate to take part in a novel therapy, helped him stick the course supplemented by support from speaking with a veteran friend  Employment and financial status:  Baseline score on the Work and Social Adjustment scale (WSAS): 22 (severe functional impairment)  Used clinic days as an opportunity to organise: ‘it gave me a challenge as such for the day so that challenge I took with as this is your day, right let’s get off your butt and sort this out because you’ve got to be there at this time and there that time […]’  Capacity to travel to 3MDR clinic:  Travel to and from the clinic (from home over 40 miles away) was via public transport, alone, and finding the clinic on the first occasion was not easy  The clinic environment:  He described his first visit to the clinic thus: ‘I was gobsmacked, I thought what am I, is this like NASA, you know, what’s going on here you know? It was just unexpected, totally unexpected because it’s such, for me it’s such a contrast between going into an old classroom or whatever […]’ | Changes recorded over time in scores on the 3MDR trial’s secondary outcome measures  WSAS Trajectory: 99,99 (missing data))  PHQ-9 Trajectory: 99,99 (missing data)  GAD-7 Trajectory: 99,99 (missing data)  MSPSS Trajectory: 99,99 (missing data)  EQ-5D-5L Trajectory:  Mobility: 99,99 (missing data)  Self-care: 99,99 (missing data)  Usual activities: 99,99 (missing data)  Pain/discomfort: 99,99 (missing data)  EQ-5D-5L Trajectory: 99,99  99,99 (missing data) |
|  | P26 (CAPS-5 trajectory during treatment period: 54,38)  Immediate treatment arm | Personality and psychopathology:  Diagnosis: PTSD, with previous trauma-focused psychological treatment without loss of PTSD diagnosis  Comorbidities: PHQ-9 baseline: 21 (severe depression), GAD-7 baseline: 16 (severe anxiety)  Severity of symptoms and presentation: CAPS-5 baseline: 54 (extreme PTSD)  Sociodemographic characteristics:  Age: 56  Education: Degree level or above  Perceived quality of life:  Baseline scores on the on the EQ-5D-5L:  Mobility: 3 (moderate problems)  Self-care: 1 (no problem)  Usual activities: 3 (moderate problems)  Pain/discomfort: 3 (moderate problems)  EQ-5D-5L Today: 40  (0=’the worst health you can imagine’, and  100=’the best health you can imagine’)  Trauma type, derived from baseline scores on the Life Events Checklist scale (LEC-5):  Learnt about and part of job transportation accident, serious accident at work, physical assault, assault with a weapon, sexual assault, other unwanted sexual assault, combat or exposure to war, captivity or kidnap, life threatening illness, severe human suffering, sudden violent death, serious injury you caused someone. Experienced childhood abuse. Witnessed fire or explosion  Attitudes and motivations:  Introduced to 3MDR by his usual therapist, who was not directly involved in the trial, who referred him in because ‘I wasn’t progressing as well as I should have been’  Expectations and experiences:  Making the journey for the first appointment was anxiety-provoking. Following a difficult weekend he rang the clinic and asked for a week off, telling his therapist it had been ‘as if the whole world had just sort of caved in’. His third treatment was followed by a difficult weekend: ‘it was unreal, you know I just blew at everything really, so I wasn’t nice to be around and the experiences were there and I just couldn’t get rid of them any more you know’. His sleep was disturbed, his memories intrusive and he felt like killing himself. Looking back over his experience he sees some good, insofar as ‘I’ve been able to talk to my family, my wife, my son about what I experienced’  Cognitive and emotional factors (Displayed words):  Words classed as having overall negative valence were much more frequent than words classed as having overall positive valence at first and this was unchanged after session 2. Positive valence words were minimal at first and also did not change  Anxiety (44%)  Negativity (20%)  Anger (11%)  Guilt (11%)  Physical NOS (5%)  Subjective unit of distress:  SUD was under 9 (extreme) at the start and dropped to above 8 (extreme) after two sessions | Exposure through walking towards trauma reminders/exercise and psychophysiological response, derived from recordings of breathing rate, heart rate and walking pace:  BRT: **60**-40  First very high then high response  (Normal BRT response to mild exercise is <30)  HRT: 85-80  Low responses to mild exercise.  Walking speed:  Just under 1m/s which is relatively slow for adults  Personal multi-sensory input (selection and sequencing of images and music, prior to and during therapy):  Finding images associated with two, separate, events three decades ago proved difficult. During the search his sleep was disturbed, and he remembered things again, but photos of a key place associated with one trauma were impossible to source online as the location had been secret. His own images were of him in his uniform, but no photos elicited strong emotions  During therapy, this meant the pictures ‘really didn’t send me right back to where I was so I think the actual feeling when I was given the treatment, I didn’t feel that I was there, you know, fully into what I was doing’  Working memory task:  Engaged in multiple tasks designed to tax working memory, by combining walking, visual tracking of a ball, calling out random numbers and scoring subjective unit of distress at the end of each image cycle. No specific measures of demand on working memory were available  SUD was under 9 (extreme) at the start and dropped to above 8 (extreme) after two sessions  Therapist coaching/relationship:  Referred by usual therapist, and returned to this person’s after dropping out of study  Number and length of sessions:  Dropped out after three treatment sessions: It wasn’t just two memories it was too much baggage and I felt I wasn’t doing myself justice or the programme, I didn’t think it was fair for your team to have me on board giving false, what I would say readings, because I’m having issues not just the two, but, so to me I would say that maybe the treatment is geared towards somebody who’s had treatment recently for their trauma rather than someone like myself who’s an old dog and had too much you know’. He was referred back to his original therapist  Cognitive engagement  Derived from recordings of breathing rate, as a proxy for more direct measures of cognitive engagement:  BRT: **60**-40  First very high then high response  (Normal BRT response to mild exercise is <30) | Relationships, home environment and social support:  Baseline score on the Multidimensional Scale for Perceived Social Support scale (MSPSS): 59 (medium support)  Before first session discussed with his family, and particularly his wife (who ‘was a little, little bit dubious because she thought I would be like a guinea pig’). His wife accompanied him to sessions.  Employment and financial status:  Baseline score on the Work and Social Adjustment scale (WSAS): 27 (severe functional impairment)  Capacity to travel to 3MDR clinic:  Travelled to Cardiff from his home in West Wales, with his wife as a passenger. The long distance was a challenge, and concentration on the drive home difficult  The clinic environment:  He described his first visit to the clinic in straightforward terms, involving trying on the harness, orienting to the building and having ‘a little try’ on the treadmill | Changes recorded over time in scores on the 3MDR trial’s secondary outcome measures  WSAS Trajectory: 27, 28 (severe to severe difficulties with work, social activities and relationships)  PHQ-9 Trajectory: 21,21 (severe to severe depression)  GAD-7 Trajectory: 16,15 (severe to severe anxiety)  MSPSS Trajectory: 59,53 (medium to medium social support).  EQ-5D-5L Trajectory:  Mobility: 3,3 (moderate problems to moderate problems)  Self-care: 1,1 (no problems to no problems)  Usual activities: 3,4 (moderate problems to severe problems)  Pain/discomfort: 3,4 (moderate problems to severe problems)  EQ-5D-5L Today Trajectory: 40,30  (0=’the worst health you can imagine’, and 100=’the best health you can imagine’) |
| **Minimal improvement** | P2 (CAPS-5 trajectory during treatment period: 50,48)  Immediate treatment arm | Personality and psychopathology:  Diagnosis: PTSD, with previous trauma-focused psychological treatment without loss of PTSD diagnosis  Comorbidities: PHQ-9 baseline: 15 (moderately severe depression), GAD-7 baseline: 12 (moderate anxiety)  Severity of symptoms and presentation: CAPS-5 baseline: 50 (extreme PTSD)  Sociodemographic characteristics:  Age: 38  Education: 1-4 GCSEs  Perceived quality of life:  Baseline scores on the on the EQ-5D-5L:  Mobility: 2 (slight problems)  Self-care: 1 (no problems)  Usual activities: 3 (moderate problems)  Pain/discomfort: 2 (slight problems)  EQ-5D-5L Today: 45  (0=’the worst health you can imagine’, and  100=’the best health you can imagine’)  Trauma type, derived from baseline scores on the Life Events Checklist scale (LEC-5):  Experienced fire explosion, transport accident, physical assault, assault with a weapon and combat or exposure to war. Witnessed transport accident, combat or exposure to war, severe human suffering, sudden violent death, sudden unexpected death of someone close to you and serious injury, harm or death.  Attitudes and motivations:  Describing his PTSD as ‘stubborn’, was open to any therapy holding out the promise of improving his life and that of his family. Had no expectations of the therapy being easy, and considered that ‘I would probably have a little bit of a rough patch, but again in the military you’re used to going through the tough to get out the other end’.  Expectations and experiences:  Had not anticipated the immersive character of the therapy, or even how the first session could pull you in: ‘It does really pull you in and I was quite surprised even from the initial sort of testing’.  He reported improvements following his therapy. These included to his sleeping, poor sleep having been a stubborn problem since 2009.  Cognitive and emotional factors (Displayed words):  Words classed as having overall negative valence were much more frequent than words classed as having overall positive valance, which substantially dipped for the last session. Positive valence words increased for the last session but still ended much lower than negative valence words.  Anxiety (30%)  Anger (18%)  Negativity (17%)  Depression (13%)  Guilt (11%)  Subjective unit of distress:  SUD was above 8 (extreme) at the start, stayed level but at the last session dropped to ± 6 (severe/moderate). | Exposure through walking towards trauma reminders/exercise and psychophysiological response, derived from recordings of breathing rate, heart rate and walking pace:  BRT: 40-40-50-**75**-xx-**60**  Starting high becoming very high response in later sessions  (Normal BRT response to mild exercise is <30)  HRT: 100-95-95-85-xx-95  Normal responses to mild exercise  Walking speed:  Around 1.1m/s which is relatively slow for adults  Personal multi-sensory input (selection and sequencing of images and music, prior to and during therapy):  He described the search for images, all from his own private collection, as ‘really, really hard’, and talked about a night of searching and selecting and telling his wife, ‘look this is so bad trying to get my experiences, ones that really affect me and move me and reduce them down to their bare bones’. Ordering images with his therapist was also difficult: ‘you know it’s one thing to look at it on a computer screen but then to hold them in your hands and to try to prioritise what was where, that’s exhausting, surprisingly so, I didn’t think it would tire me that much’. In contrast, selecting music was easier, and included a track current during a tour followed by ‘one of the songs that I sing in the car with the kids when we’re having fun’  Images moved out over sessions, with those ‘at the top of scale’ featuring at the end of each visit and images provoking no reaction dropping out altogether. Decisions on selections were made at the end of sessions in anticipation of the next visit  Working memory task:  Engaged in multiple tasks designed to tax working memory, by combining walking, visual tracking of a ball, calling out random numbers and scoring subjective unit of distress at the end of each image cycle. No specific measures of demand on working memory were available  SUD was above 8 (extreme) at the start, stayed level but at the last session dropped to ± 6 (severe/moderate)  Therapist coaching/relationship:  Introduced to 3MDR by his regular therapist, whose view he trusted. Knowing his treating therapist in advance was helpful, and had he started with someone new ‘it probably would’ve had to have been a longer process’  Number and length of sessions:  He would have had more sessions had that been the offer at the start, but would not have added sessions mid-way through  Cognitive engagement  Derived from recordings of breathing rate, as a proxy for more direct measures of cognitive engagement:  BRT: 40-40-50-**75**-xx-**60**  Starting high becoming very high response in later sessions  (Normal BRT response to mild exercise is <30) | Relationships, home environment and social support:  Baseline score on the Multidimensional Scale for Perceived Social Support scale (MSPSS): 68 (high support)  He discussed his treatment with his wife, and never thought of quitting: ‘I don’t quit anything. I’m still very military-minded’.  Employment and financial status:  Baseline score on the Work and Social Adjustment scale (WSAS): 20 (significant functional impairment)  Fitting treatment into his life was possible as he was able to adapt his working hours to fit around his employment (with disabled children), but made for very long days with lengthy drives.  Capacity to travel to 3MDR clinic:  Long days with lengthy drives. An unwanted effect was of loss of attention and poor concentration following sessions, so that the detail of drives to work or home could not be recalled.  The clinic environment:  He had prior experience of gait analysis rooms, and ‘was impressed with the set-up’ | Changes recorded over time in scores on the 3MDR trial’s secondary outcome measures  WSAS Trajectory: 20,27 (significant to severe difficulties with work, social activities and relationships)  PHQ-9 Trajectory: 15,16 (moderately severe to moderately severe depression)  GAD-7 Trajectory: 12,16 (moderate to severe anxiety)  MSPSS Trajectory: 68,63 (high to high social support)  EQ-5D-5L Trajectory:  Mobility: 2,3 (slight problems to moderate problems)  Self-care: 1,1 (no problems to no problems)  Usual activities: 3,3 (moderate problems to moderate problems)  Pain/discomfort: 2,2 (slight problems to slight problems)  EQ-5D-5L Today Trajectory: 45,55  (0=’the worst health you can imagine’, and 100=’the best health you can imagine’) |
|  | P25 (CAPS-5 trajectory during treatment period: 30,29)  Immediate treatment arm | Personality and psychopathology:  Diagnosis: PTSD, with previous trauma-focused psychological treatment without loss of PTSD diagnosis  Comorbidities: PHQ-9 baseline: 6 (mild depression), GAD-7 baseline: 5 (mild anxiety)  Severity of symptoms and presentation: CAPS-5 baseline: 30 (moderate PTSD)  Sociodemographic characteristics:  Age: 55  Education: NVQ level 4-5  Perceived quality of life:  Baseline scores on the on the EQ-5D-5L:  Mobility: 3 (moderate problems)  Self-care: 2 (slight problems)  Usual activities: 3 (moderate problems)  Pain/discomfort: 4 (severe problems)  EQ-5D-5L Today: 50  (0=’the worst health you can imagine’, and  100=’the best health you can imagine’)  Trauma type, derived from baseline scores on the Life Events Checklist scale (LEC-5):  Experienced natural disaster, fire or explosion, transportation accident, serious accident at work, exposure to toxic chemicals, physical assault, sexual assault, captivity or kidnap, life threatening illness, severe human suffering, childhood physical abuse, and childhood sexual abuse. Part of my job is combat or exposure to war, sudden violent death, and sudden unexpected death of someone close to you  Attitudes and motivations:  Ahead of having 3MDR he was ‘shit scared, plain and simple, you know, and I made that known to [name of therapist] as well, that I was you know, shit scared of what it might bring up’. He entered therapy with ‘a badge of shame attached to me, and guilt’  Expectations and experiences:  ‘I wanted to give it a 110%, I know that I’m damaged and if I could get some help myself then I wanted that and again you know, let’s see what happens over the year’  Cognitive and emotional factors (Displayed words):  Words classed as having overall negative valence were slightly more frequent than words classed as having overall positive valence at first, rapidly dropping to end substantially reduced. Positive valence words were similar to negative valence words at first and gradually increased to a peak midway, but then rapidly declined to a low number, slightly lower than negative valence words  Positivity (54%)  Anger (15%)  Depression (9%)  Negativity (7%)  Physical NOS (6%)  Note:  Anxiety only 2%  Subjective unit of distress:  SUD was below 7 (severe) at the start, gradually dropped to ± 5 (moderate) but rose to 7 (severe) again at the end of 5 sessions | Exposure through walking towards trauma reminders/exercise and psychophysiological response, derived from recordings of breathing rate, heart rate and walking pace:  BRT: 30-35-**60**-15-50  First two moderately high, midway very high response, last but one low, final is high response  (Normal BRT response to mild exercise is <30)  HRT: 95-90-95-95-95  Normal response to mild walking exercise  Walking speed:  Around 0.75 m/s which is very slow for adults  Personal multi-sensory input (selection and sequencing of images and music, prior to and during therapy):  Searching for images, with therapist help, was a revelation. Alongside finding online, ‘difficult’, pictures he also found a ‘top of the mountain’ document, the existence of which he had not previously been aware, which recorded an official judgment that the trauma he had been involved in was down to another person’s error and not to his negligence. This had been the position taken by investigators at the time. Finding this was a tearful event, which ‘sent me on a huge roller coaster of emotions’, and this single ‘document took over so much’. His music ‘was a godsend really’, both being important and selected because they were very different and because both words and music were meaningful  Working memory task:  Engaged in multiple tasks designed to tax working memory, by combining walking, visual tracking of a ball, calling out random numbers and scoring subjective unit of distress at the end of each image cycle. No specific measures of demand on working memory were available  SUD was below 7 (severe) at the start, gradually dropped to ± 5 (moderate) but rose to 7 (severe) again at the end of 5 sessions  Therapist coaching/relationship:  His 3MDR therapist was new to him, and his referring therapist’s recommendation was important  Number and length of sessions:  His view was that there does not need to be a set number of sessions ‘because I think for each person it could be different’  Cognitive engagement  Derived from recordings of breathing rate, as a proxy for more direct measures of cognitive engagement:  BRT: 30-35-**60**-15-50  First two moderately high, midway very high response, last but one low, final is high response  (Normal BRT response to mild exercise is <30) | Relationships, home environment and social support:  Baseline score on the Multidimensional Scale for Perceived Social Support scale (MSPSS): 38 (medium support)  Says his circle of friends is small, so talked about joining the study and preparation with a charity welfare officer. Talking with, and thinking about, his mates helped him to keep coming to the clinic along with trying to reach a sense of closure:  Employment and financial status:  Baseline score on the Work and Social Adjustment scale (WSAS): 19 (significant functional impairment)  Accommodation of 3MDR into everyday life not discussed  Capacity to travel to 3MDR clinic:  His friends were important, travelling with him to the clinic  The clinic environment:  He saw the location of services for veterans as important: ‘friendly to the eye, friendly to the smell, friendly to the touch’, and his first clinic visit involved taking the set-up in: ‘so it’s going to be up there and you’re going to walk so I was, going through the motions I suppose’  The environment was not to his liking: ‘the [xxx] Hospital itself is just a machine and a lot of people are like robots, and I mean that in all honesty, and I found it exceptionally difficult’ | Changes recorded over time in scores on the 3MDR trial’s secondary outcome measures  WSAS Trajectory: 19,36 (significant to severe difficulties with work, social activities and relationships)  PHQ-9 Trajectory: 6,11 (mild to moderate depression)  GAD-7 Trajectory: 5,11 (mild to moderate anxiety)  MSPSS Trajectory: 38,33 (medium to medium social support).  EQ-5D-5L Trajectory:  Mobility: 3,4 (moderate problems to severe problems)  Self-care: 2,3 (slight problems to moderate problems)  Usual activities: 3,3 (moderate problems to moderate problems)  Pain/discomfort: 4,4 (severe problems to severe problems)  EQ-5D-5L Today Trajectory: 50,55  (0=’the worst health you can imagine’, and 100=’the best health you can imagine’) |
| Key: PTSD=Post-Traumatic Stress Disorder; CAPS-5=Clinician Administered Post Traumatic Stress Scale; LEC-5=Life Events Checklist for DSM-5; PHQ-9=Patient Health Questionnaire; GAD-7=Generalised Anxiety Disorder Questionnaire; MSPSS=Multidimensional Scale for Perceived Social Support; WSAS=Work and Social Adjustment Scale; EQ-5D-5L= The EuroQol Five-Dimensional Descriptive System; 99=missing data; SUD=subjective unit of distress; BRT=breathing rate; HRT=heart rate; NVQ=National Vocational Qualification (post-16 education qualification); GCSE=General Certificate of Secondary Education or equivalent (education qualification usually completed at age 16); NOS=not otherwise specified.  EQ-5D-5L anxiety/depression scores not used in favour of GAD-7 and PHQ-9. | | | | | |
